# Supplementary material for: Stress-induced ordering evolution of 1D segmented heteronanostructures and their chemical post-transformations
Source: Nat Commun. 2024 Apr 13;15:3208. doi: 10.1038/s41467-024-47446-7 (PMC11271508; doi:10.1038/s41467-024-47446-7)
Supplement: Supplementary file 1 — Supplementary Information [file 41467_2024_47446_MOESM1_ESM.pdf]

Supplementary Information

Stress-induced ordering evolution of 1D segmented heteronanostructures and their chemical post-transformations

Qing-Xia Chen⋆, Yu-Yang Lu⋆, Yang Yang⋆, Li-Ge Chang, Yi Li, Yuan Yang, Zhen He, Jian-Wei Liu*, Yong Ni* and Shu-Hong Yu*

⋆These authors contributed equally to this work.

*Corresponding author: Jian-Wei Liu (jwliu13@ustc.edu.cn), Yong Ni (yni@ustc.edu.cn) or Shu-Hong Yu (shyu@ustc.edu.cn)

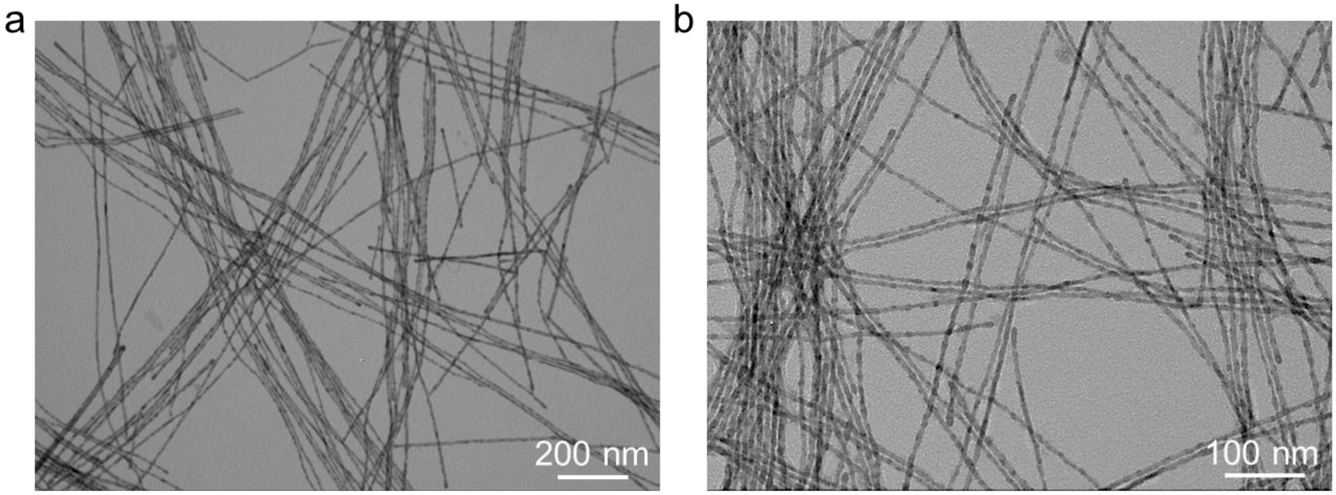

Supplementary Figure 1 | Large-scale TEM images of Te/Ag_2Te SHs. a, TEM image of Te/Ag_2Te SHs with a scale bar of 200 nm. **b,** TEM image of Te/Ag_2Te SHs with a scale bar of 100 nm.

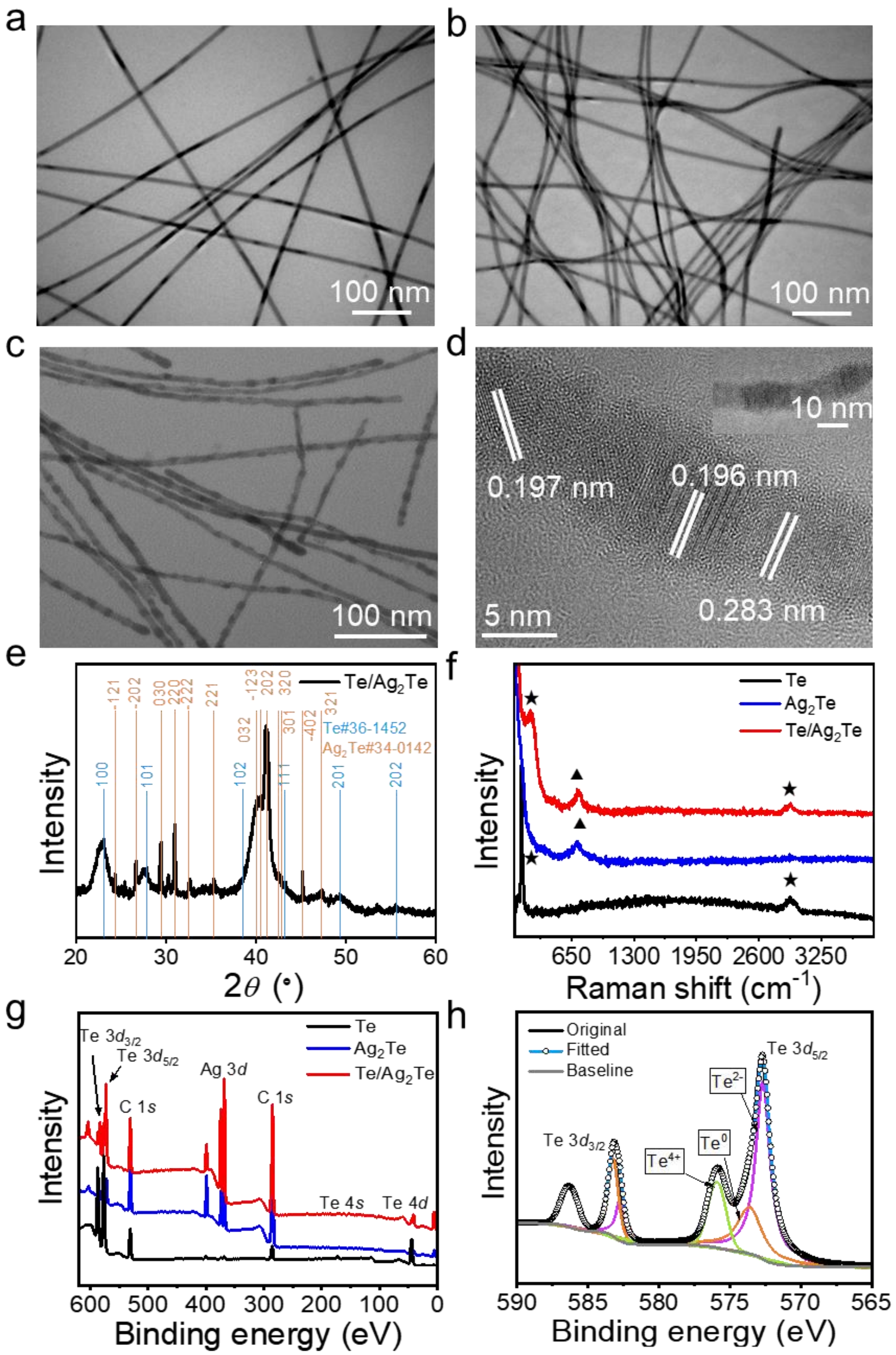

Supplementary Figure 2 | Morphological and structural characterizations of Te NWs, Ag_2Te NWs and Te/Ag_2Te SHs. a, b, c, TEM images of Te, Ag_2Te, and Te/Ag_2Te. **d,** HRTEM images of Te/Ag_2Te SHs, with spacings of 0.197 and 0.283 nm corresponding to Te and Ag_2Te, respectively. Inset: HRTEM image of Te/Ag_2Te SHs. This observation potently establishes that the heterogeneous NW contained Te and Ag_2Te phase alternations along its axes. **e,** XRD pattern analysis of Te/Ag_2Te SHs. **f,** The survey XPS spectra, showing the existing Ag element in Te/Ag_2Te. **g,** Fitted XPS spectra of Te 3*d* orbital, showing the presence of both Te^0 and Te^{2-} phases. **h,** Raman spectra of Te/Ag_2Te SHs. Source data are provided as a Source Data file.

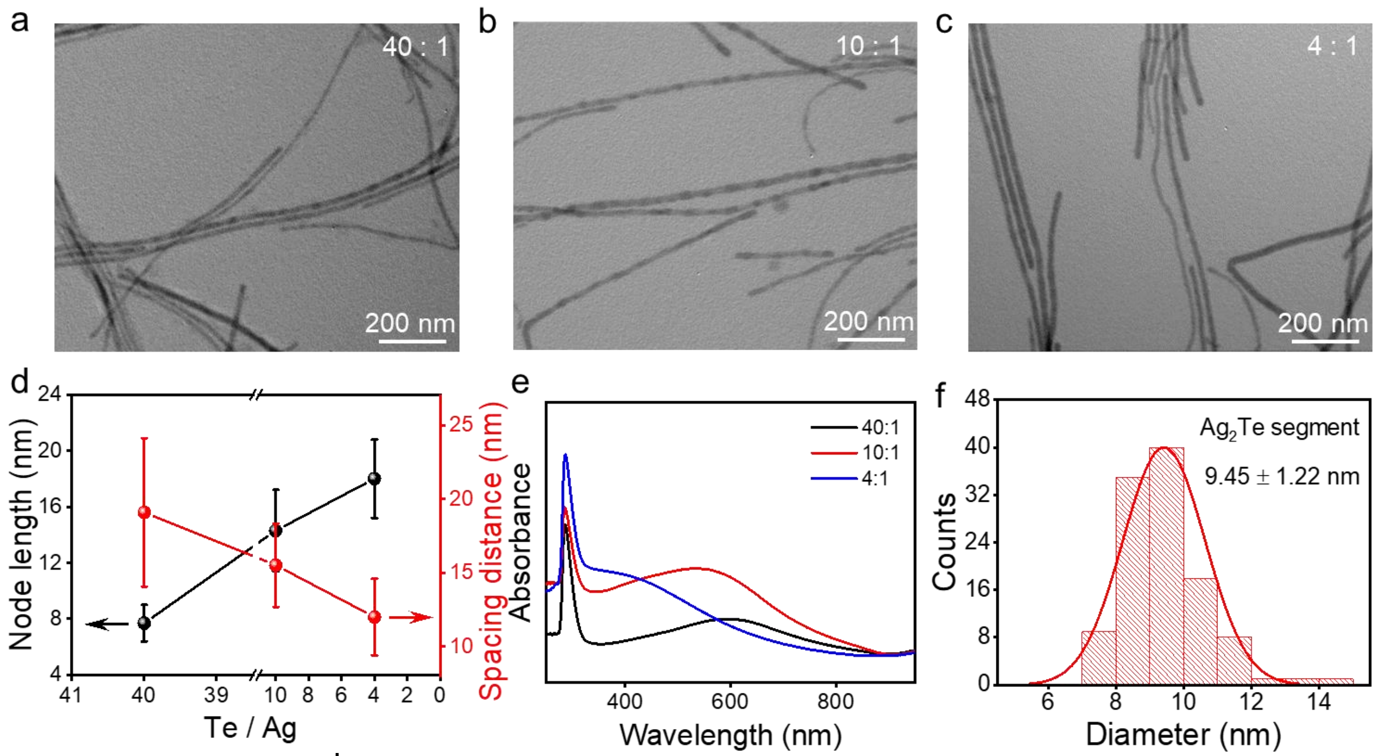

Supplementary Figure 3 | Morphological and structural characterizations Te/Ag_2Te SHs with various ratios of Ag/Te. a, b, c, TEM images of Te/Ag_2Te with Ag/Te=40, 10 and 4, showing the Ag_2Te segments lengthen as Ag^+ increases. **d,** Segment length and separation variations as the Te/Ag ratio changes. For calculation, the segment length and separation variations were measured three times. **e,** UV–vis spectra, showing the peak at 600 nm in Te disappears accompanied by a shoulder peak at 400 nm in Ag_2Te with the increasing Ag^+. **f,** Histogram of diameter distribution of Ag_2Te segments in Te/ Ag_2Te SHs with Te/Ag=10. The volume expansion from Te to Ag_2Te is about 10%. Source data are provided as a Source Data file.

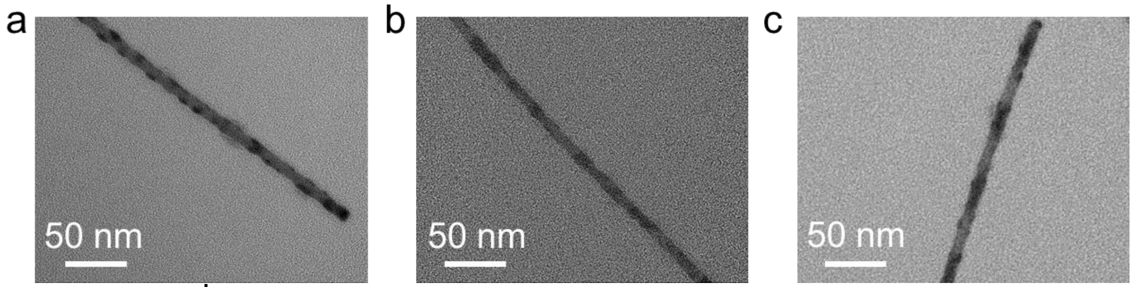

Supplementary Figure 4 | Time-resolved TEM images of Te/Ag_2Te SHs. a, Random distributed islands. **b,** Irregular stripes. **c,** Periodic segmented structure.

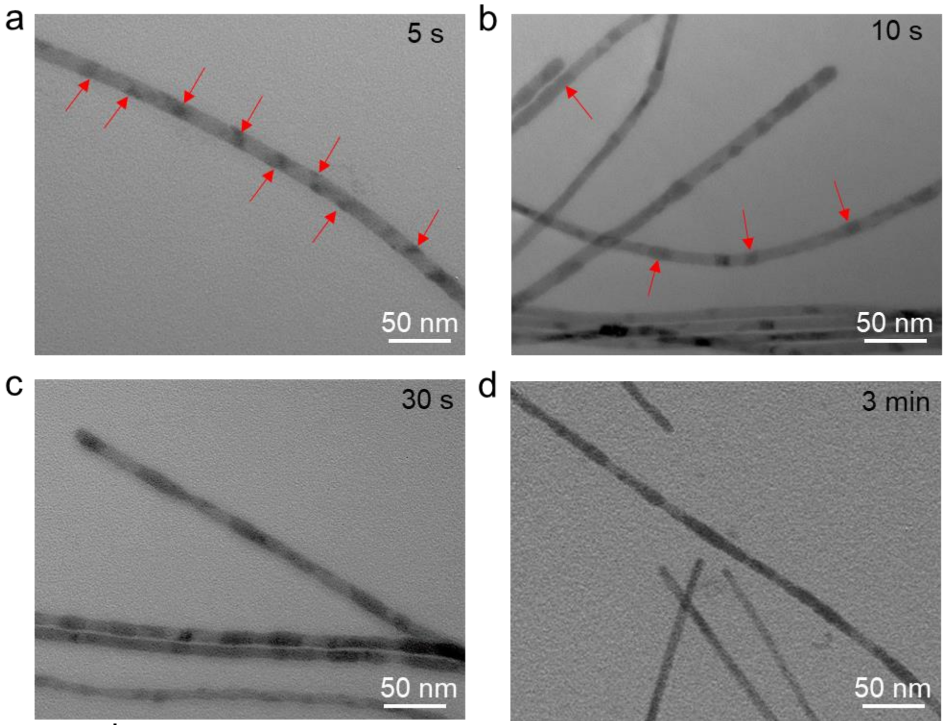

Supplementary Figure 5 | Morphological evolutions of Te/Ag_2Te SHs. a, b, c, d, TEM images captured at 5 s, 10 s, 30 s, and 3 min in the reaction of Ag^+ and Te with Ag/Te =1. Te NWs were randomly dotted with small islands at 5 s, which started to grow radially throughout the whole NW at 10 s and developed into disordered SHs at 30 s. And at 3 min, the ordered SHs evolving from disordered one can be observed. The red arrows represent the islands formed in the initial stage and their later evolution into the striped structure, respectively.

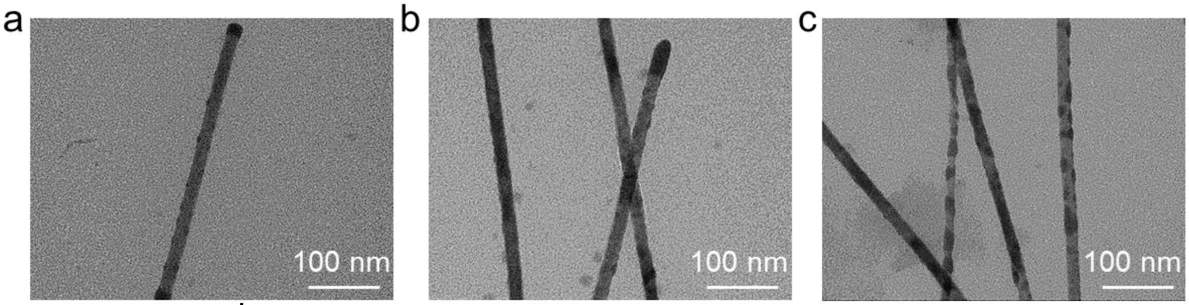

Supplementary Figure 6 | Morphological characterizations of Te/Ag_2Te SHs at various times under ice bath. TEM images of Te/Ag_2Te SHs captured at (**a**) 2 s, (**b**) 10 s, and (**c**) 1 min under ice bath. Ice bath was used to further decelerate the reaction. The initial Ag_2Te islands embedded sporadically into Te NW templates with very shallow depth and small size. As the reaction progressed, the islands grew larger and more numerous. And these islands continued to develop and some islands begun to permeate throughout the diameter, forming the stripped structures.

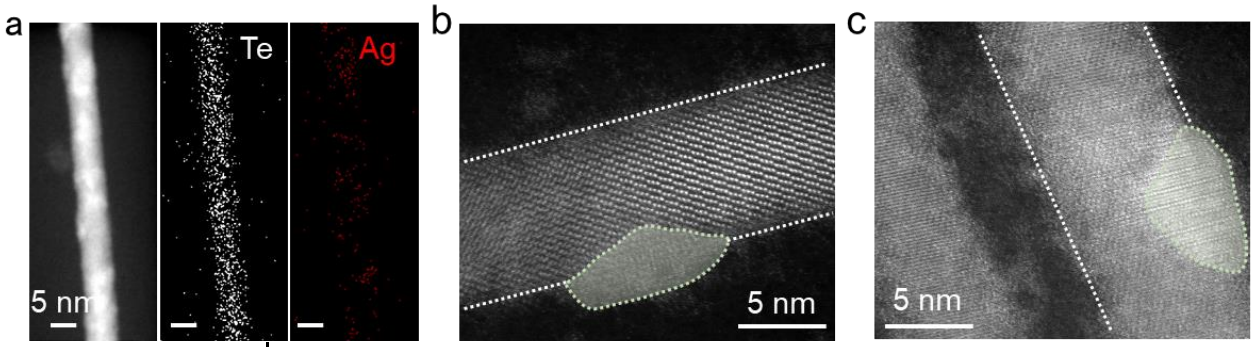

Supplementary Figure 7 │ Detailed characterization of initial islands. a, HADDF image and EDS mapping of Te and Ag. All scale bars are 5 nm. **b, c,** HADDF images of islands on NW.

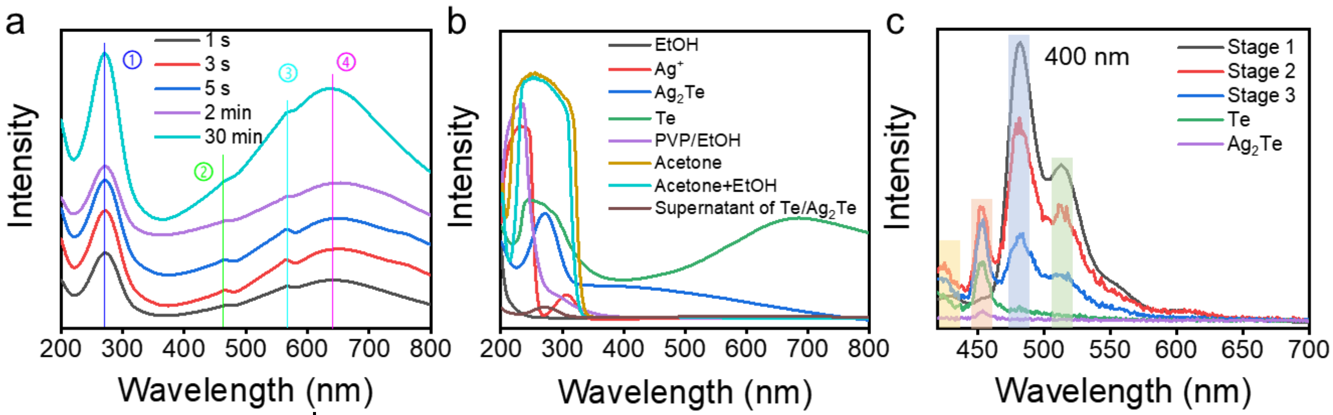

Supplementary Figure 8 │ UV−vis absorption and fluorescence emission spectra of Te/Ag_2Te SHs at various stages. a, UV−vis absorption spectra of various stages of Te/Ag_2Te SHs. **b,** UV−vis absorption spectra of Te, Ag_2Te NWs and other solutions. **c,** Fluorescence emission spectra of Te/Ag_2Te captured at various times under ice bath, Te NWs and Ag_2Te NWs. When Ag^+ is added to Te NWs in EG, two shoulder peaks appear at wavelengths of 464 and 566 nm in addition to the absorption peaks of templates. These two new absorption peaks result from the formation of heterostructures between Te and Ag_2Te. This can be confirmed by the fluorescent emission spectra under excitation of 400 nm, where the emission peak at 454 nm of Ag_2Te gradually increases with the evolution from island to segmented structures. Source data are provided as a Source Data file.

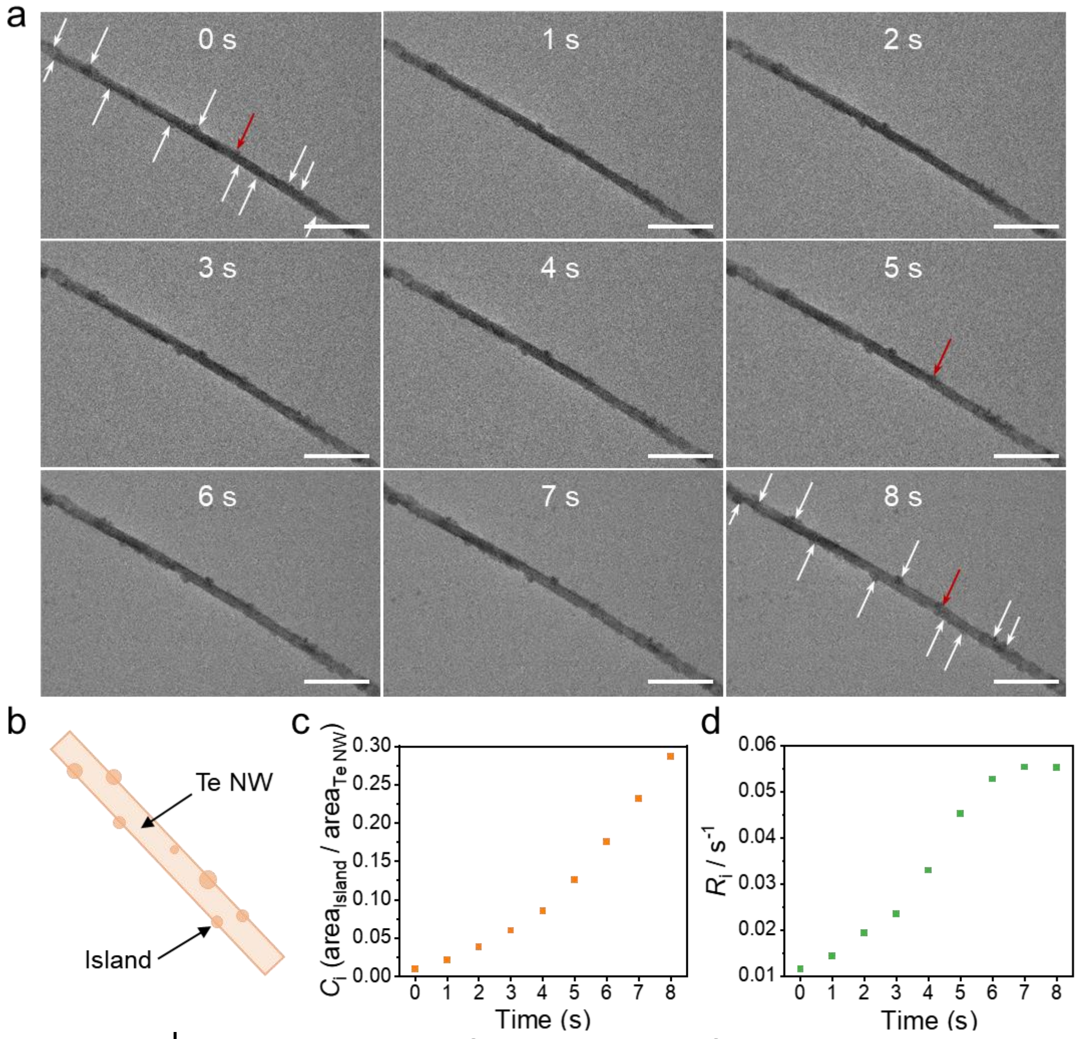

Supplementary Figure 9 | *In-situ* observation of the initial island forming. a, Time sequential TEM images of island forming. Scale bars in all panels are 100 nm. **b,** Illustration of the calculation of island area. **c,** Scatter diagram of island area compared to Te NW area, C_i. **d,** Corresponding rates of island forming areas, R_i. Source data are provided as a Source Data file.

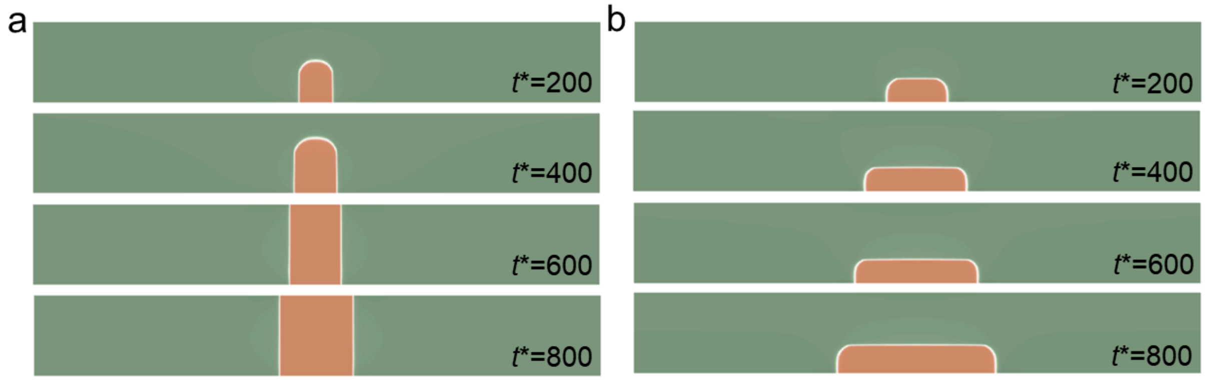

Supplementary Figure 10 | Effect of anisotropic interfacial energy on the growth of Ag_2Te island without considering the stress at different dimensionless times (t^*=200, 400, 600, 800). a, The anisotropic interfacial energy parameter $\kappa x/\kappa y$=1/4. **b,** The anisotropic interfacial energy parameter κ_x/κ_y=4.

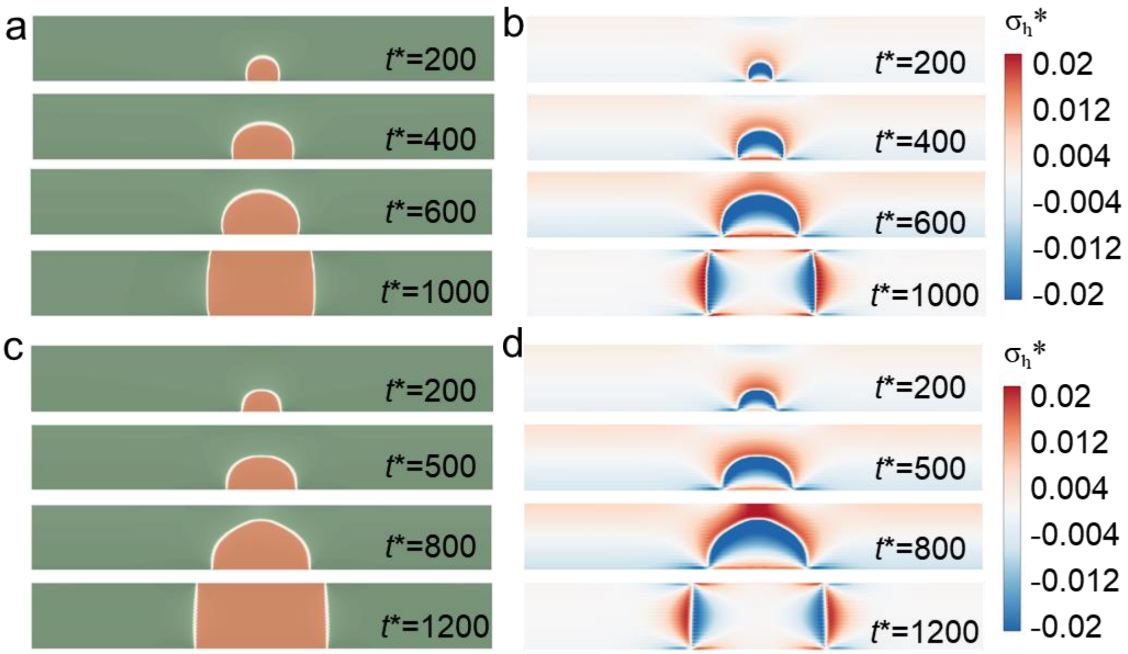

Supplementary Figure 11 │ Effects of anisotropic interfacial energy and mismatch strain on the growth of Ag_2Te island. a, b, The morphology of Ag_2Te island at different dimensionless times (t^*=200, 400, 600, 1000) and the corresponding distribution of dimensionless hydrostatic stress when the anisotropic interfacial energy parameter κ_x/κ_y is set as 1/4, and the expansion coefficient is set as 0.04. **c, d,** The morphology of Ag_2Te island at different dimensionless times (t^*=200, 500, 800, 1200) and the corresponding distribution of dimensionless hydrostatic stress when the anisotropic interfacial energy parameter κ_x/κ_y is set as 4, and the expansion coefficient is set as 0.04.

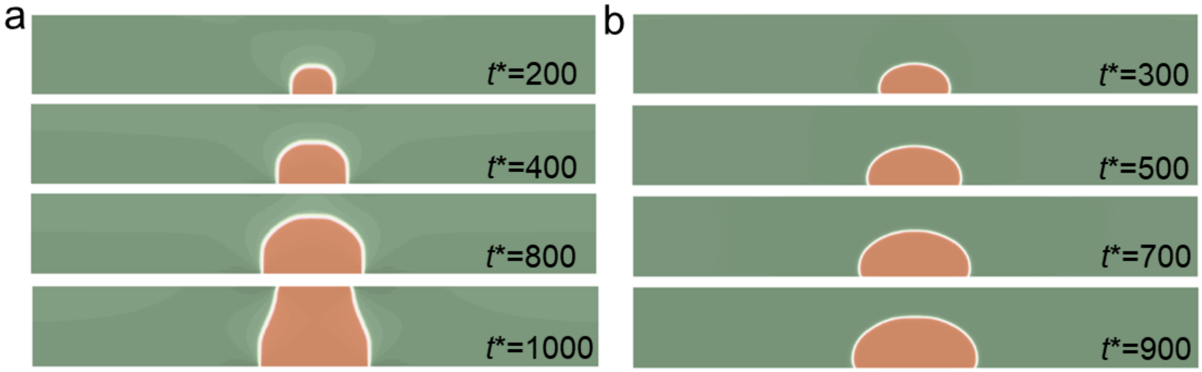

Supplementary Figure 12 │ Effects of interfacial diffusion and surface diffusion on the growth of Ag_2Te island. a, The morphology evolution of Ag_2Te island when considering the effect of interfacial diffusion at different dimensionless times (t^*=200, 400, 800, 1000). **b,** The morphology evolution of Ag_2Te island considering fast surface diffusion coefficient (Ds =2.5×10^{-9}cm^2 s^{-1}) at different dimensionless times (t^*=200, 400, 800, 1000).

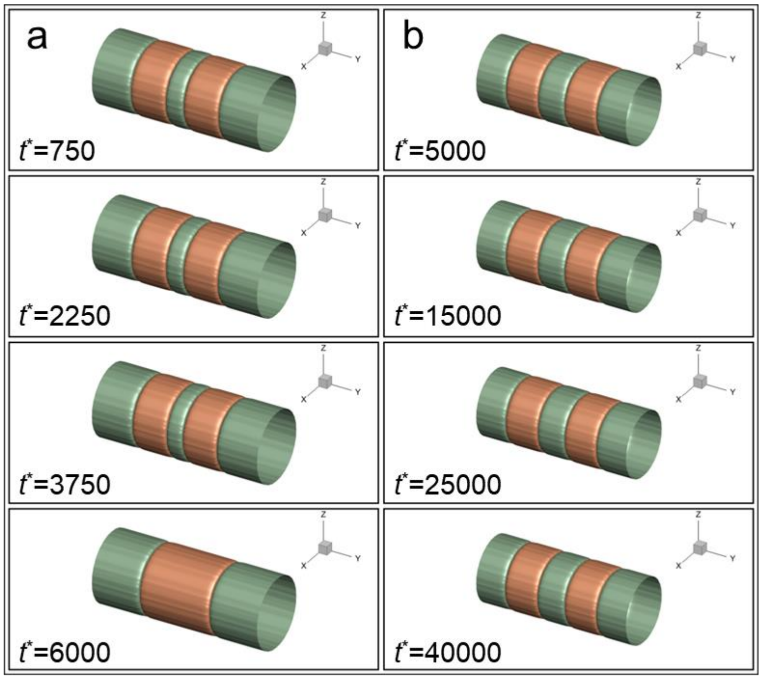

Supplementary Figure 13 | Dimensionless concentration evolution results of the two segments of the same size. a, When the segments are very close to each other at times t^*=750, t^*=2250, t^*=3750 and t^*=6000. **b,** When the segments are far from each other at times t^*=5000, t^*=15000, t^*=25000 and t^*=40000.

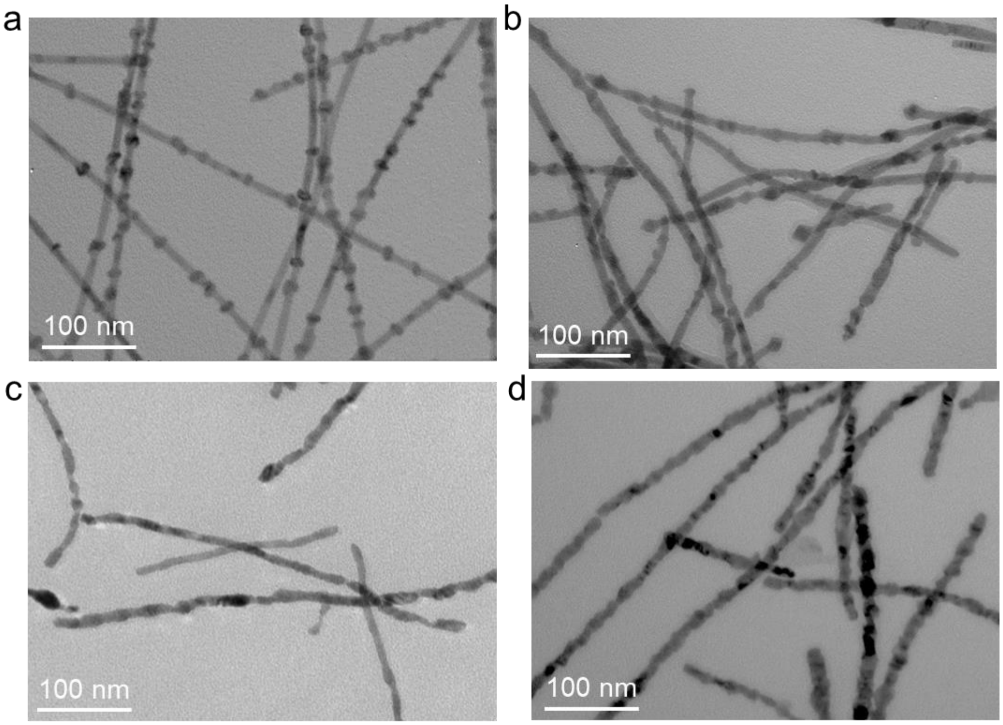

Supplementary Figure 14 | Typical TEM images of as-transformed G.1 SHs. a, Te/PbTe. **b,** Te/$Cu_{1.75}$Te. **c,** Te/Bi_2Te_3. **d,** Te/CdTe.

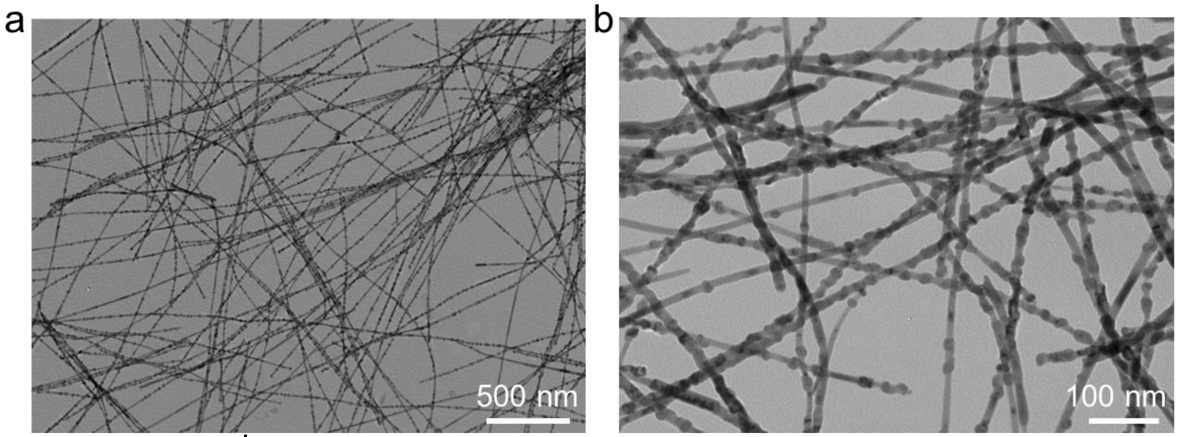

Supplementary Figure 15 │ Large-scale TEM images of Te/PbTe SHs. a, TEM image of Te/PbTe SHs with a scale bar of 500 nm. **b,** TEM image of Te/PbTe SHs with a scale bar of 100 nm.

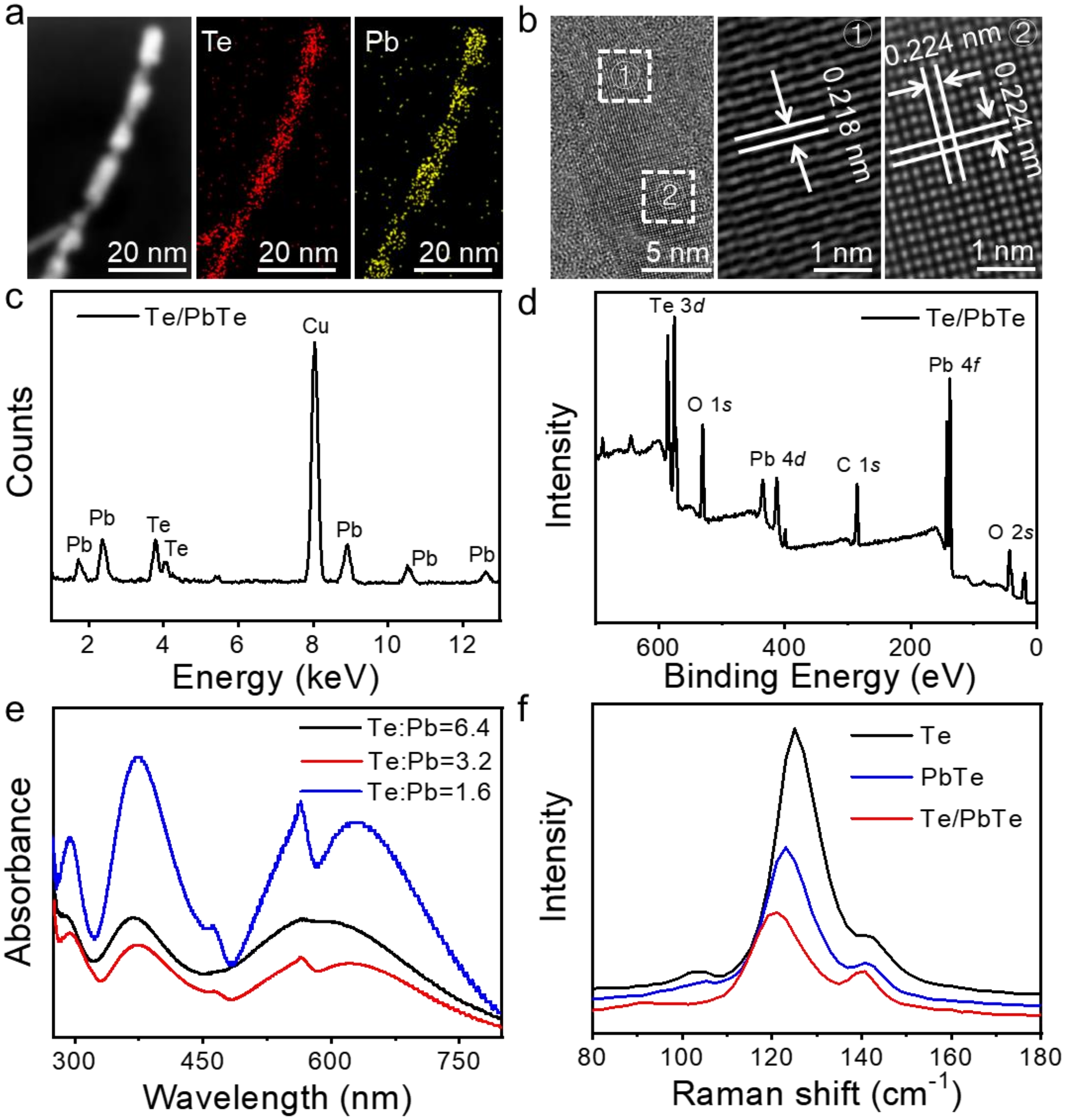

Supplementary Figure 16 | Morphological and structural characterizations Te/PbTe SHs. a, HADDF-STEM and elemental mapping characterizations, showing Te distributed homogeneously while Pb heterogeneously distributed along the NW. **b,** HRTEM images, showing the spacings of 0.218 and 0.224 nm corresponding to Te and PbTe, respectively. **c,** EDS. **d,** The survey XPS spectra, showing the existence of Te and Pb. **e,** UV–vis spectra, showing a sharp peak at 580 nm increasingly defined with increasing Pb^{2+}. **f,** Raman spectra, showing the downshift of the peak at 125 nm in Te/PbTe. Source data are provided as a Source Data file.

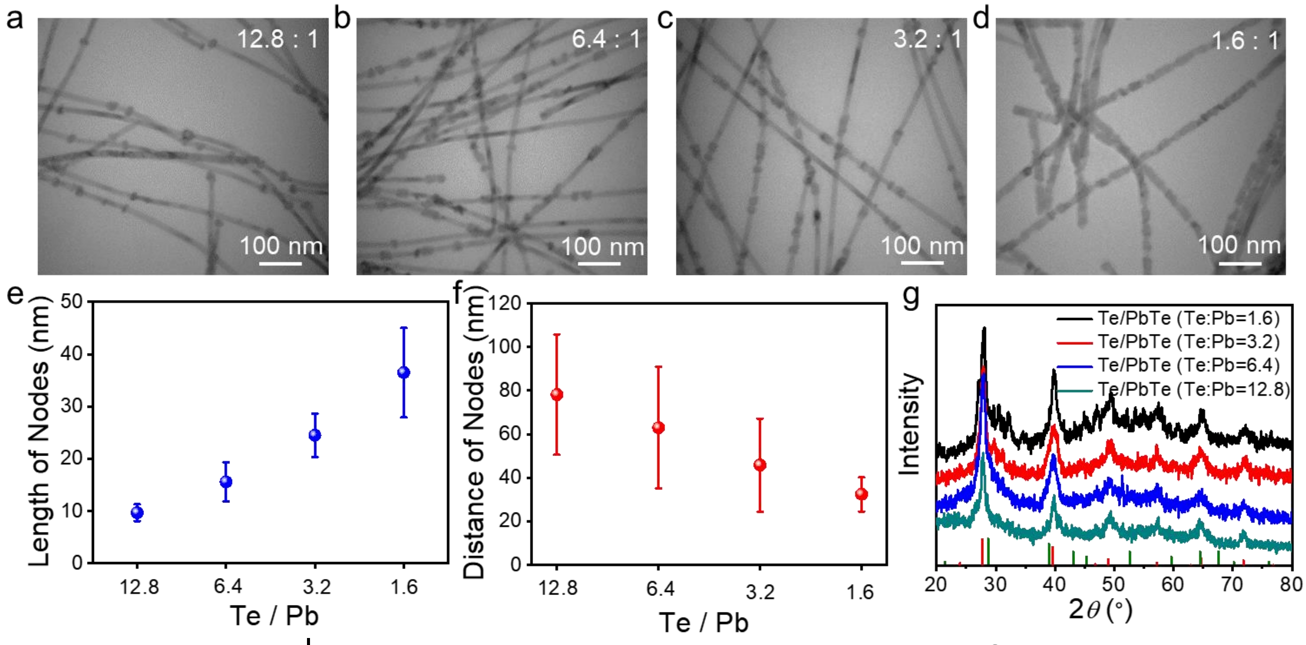

Supplementary Figure 17 │ Morphological and structural characterizations of Te/PbTe SHs with various ratios of Pb/Te. a, b, c, d, TEM images with Pb/Te=12.8, 6.4, 3.2 and 1.6. **e, f,** Length and distance of PbTe segments evolutions with increasing Pb^{2+}. Error bars correspond to the standard deviations taken over three measurements. **g,** XRD pattern. Source data are provided as a Source Data file.

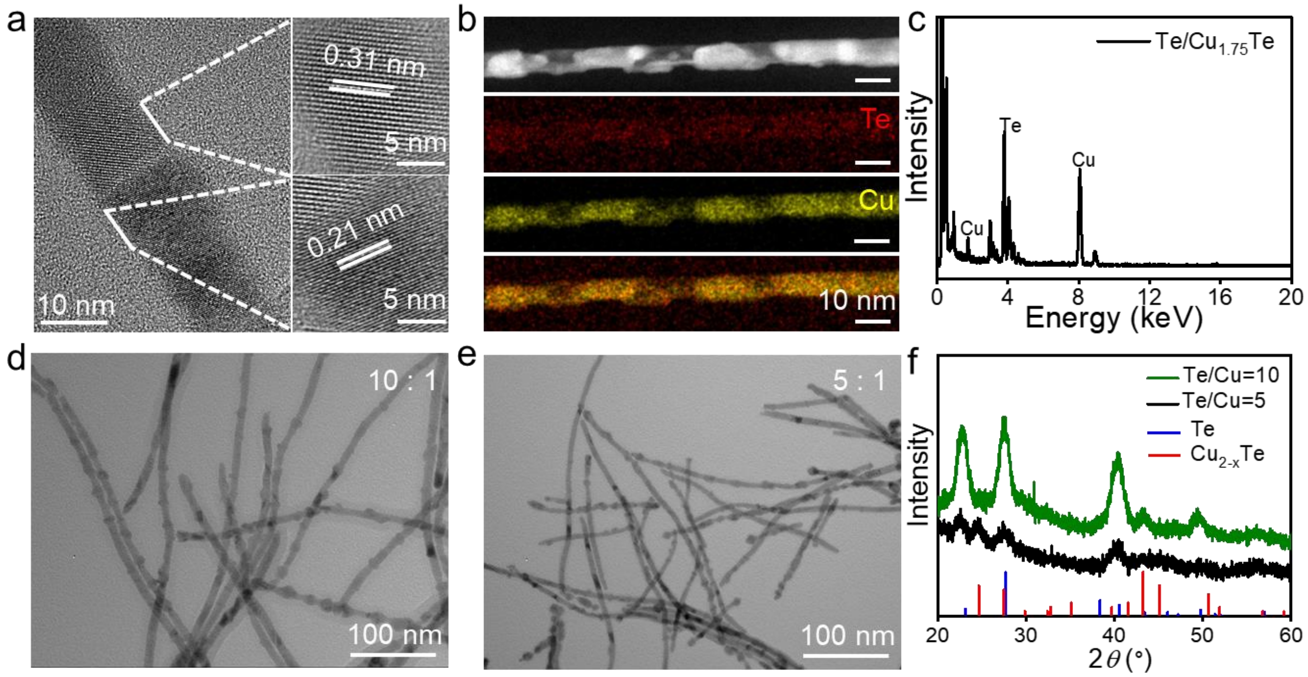

Supplementary Figure 18 │ Morphological and structural characterizations of Te/$Cu_{1.75}$Te SHs. a, HRTEM images with spacings of 0.31 and 0.21 nm corresponding to Te and $Cu_{1.75}$Te, respectively. **b,** HADDF-STEM and elemental mapping characterizations. All scale bar are 10 nm. **c,** EDS. **d, e,** TEM images with Cu/Te=10 and 5. **f,** XRD pattern of Te/ $Cu_{1.75}$Te with different quantities of Cu precursor, showing the peak at 24.4° attributed to $Cu_{1.75}$Te disappears as Cu precursor reduces. Source data are provided as a Source Data file.

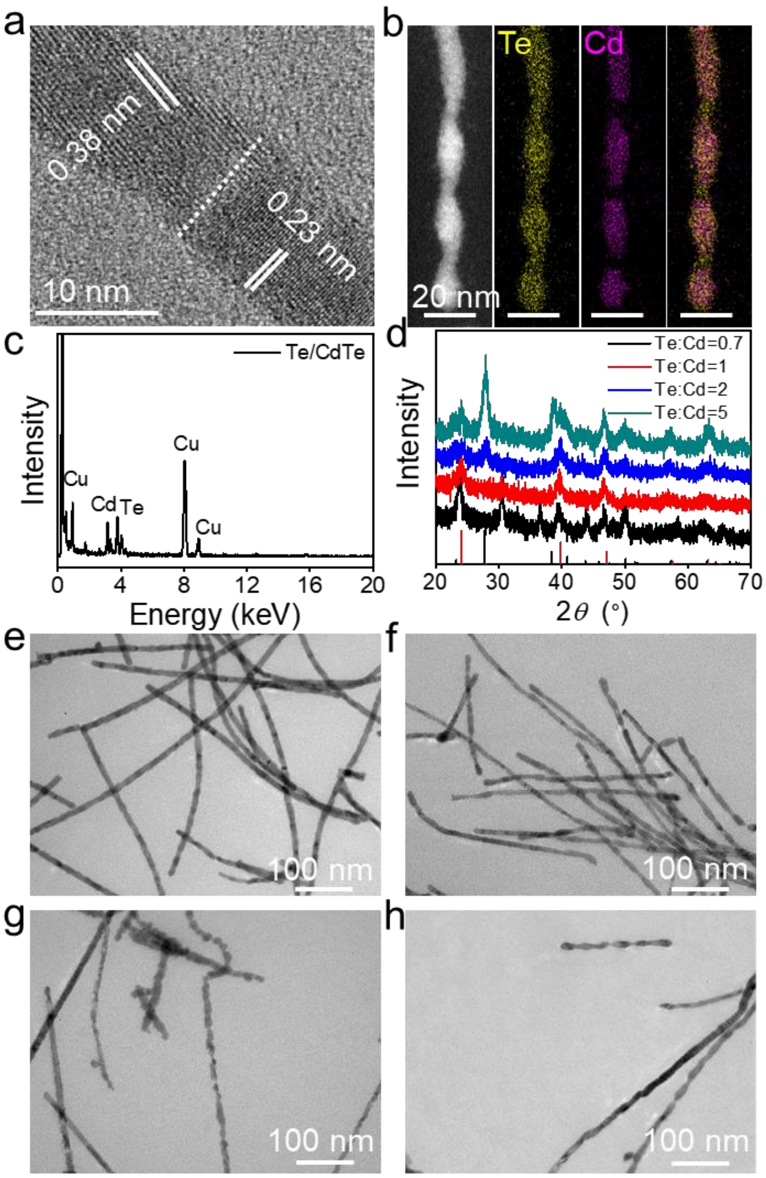

Supplementary Figure 19 | Morphological and structural characterizations of Te/CdTe SHs. a, HRTEM image with spacings of 0.38 and 0.23 nm corresponding to Te and $Cu_{1.75}Te$, respectively. **b,** HADDF-STEM and elemental mapping characterizations. All scale bar are 10 nm. **c,** EDS. **d,** XRD patterns with different quantities of Cd precursor, showing the peak at 27.9° attributed to Te weakens accompanied by the peak at 24° attributed to CdTe strengthening as Cd precursor increases. **e, f, g, h,** TEM images with Te/Cd=0.7, 1, 2 and 5. Source data are provided as a Source Data file.

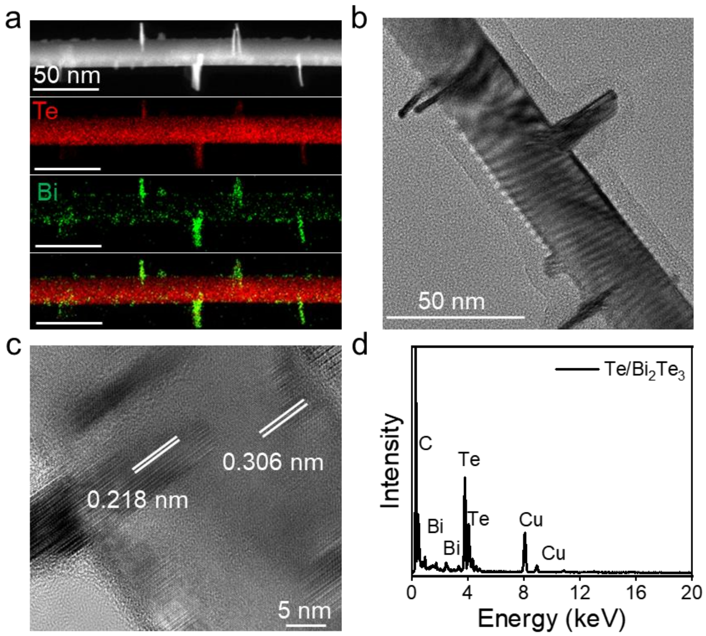

Supplementary Figure 20 | Morphological and structural characterizations of Te/Bi_2Te_3 SHs. a, HADDF-STEM and elemental mapping characterizations. All scale bar are 50 nm. **b, c,** HRTEM images with spacings of 0.306 and 0.218 nm corresponding to Te and Bi_2Te_3, respectively. **d,** EDS. Source data are provided as a Source Data file.

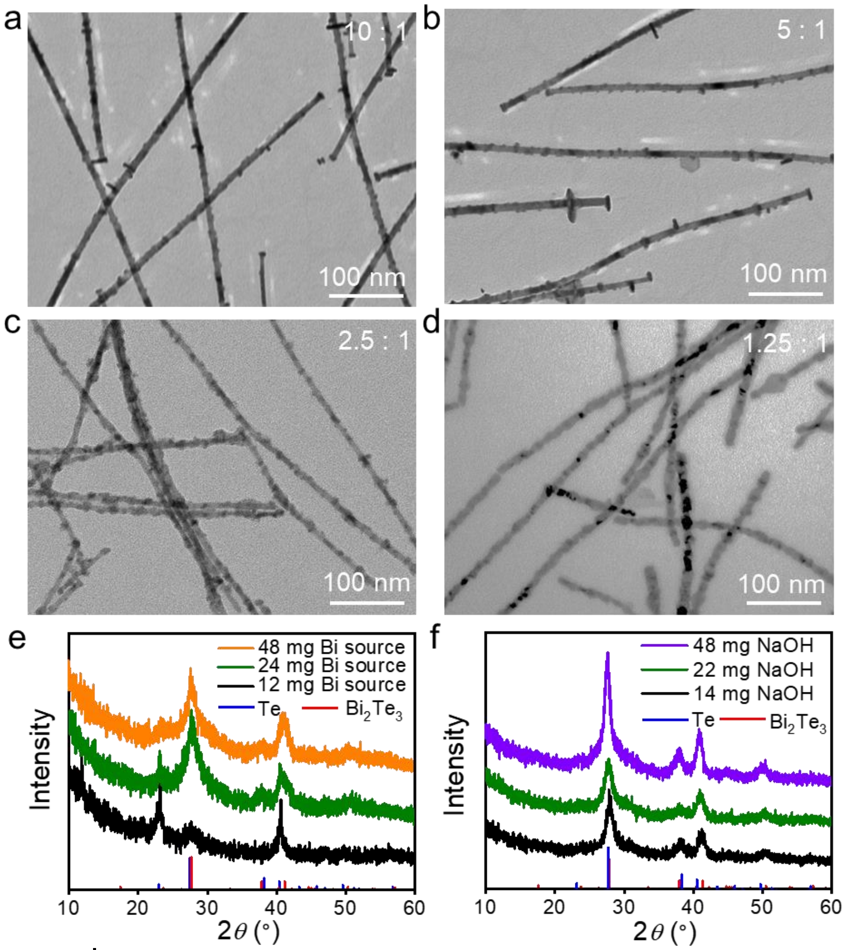

Supplementary Figure 21 | Morphological and structural characterizations of Te/Bi_2Te_3 SHs with various ratios of Bi/Te. a, b, c, d, TEM images with Bi/Te=10, 5, 2.5 and 1.25. **e, f,** XRD patterns of Te/Bi_2Te_3 with different quantities of Bi precursor and NaOH. The peak at 23.2° attributed to Te weakens with the increasing Bi source, while the peak at 40.9° attributed to Bi_2Te_3 strengthens with the increasing NaOH. Source data are provided as a Source Data file.

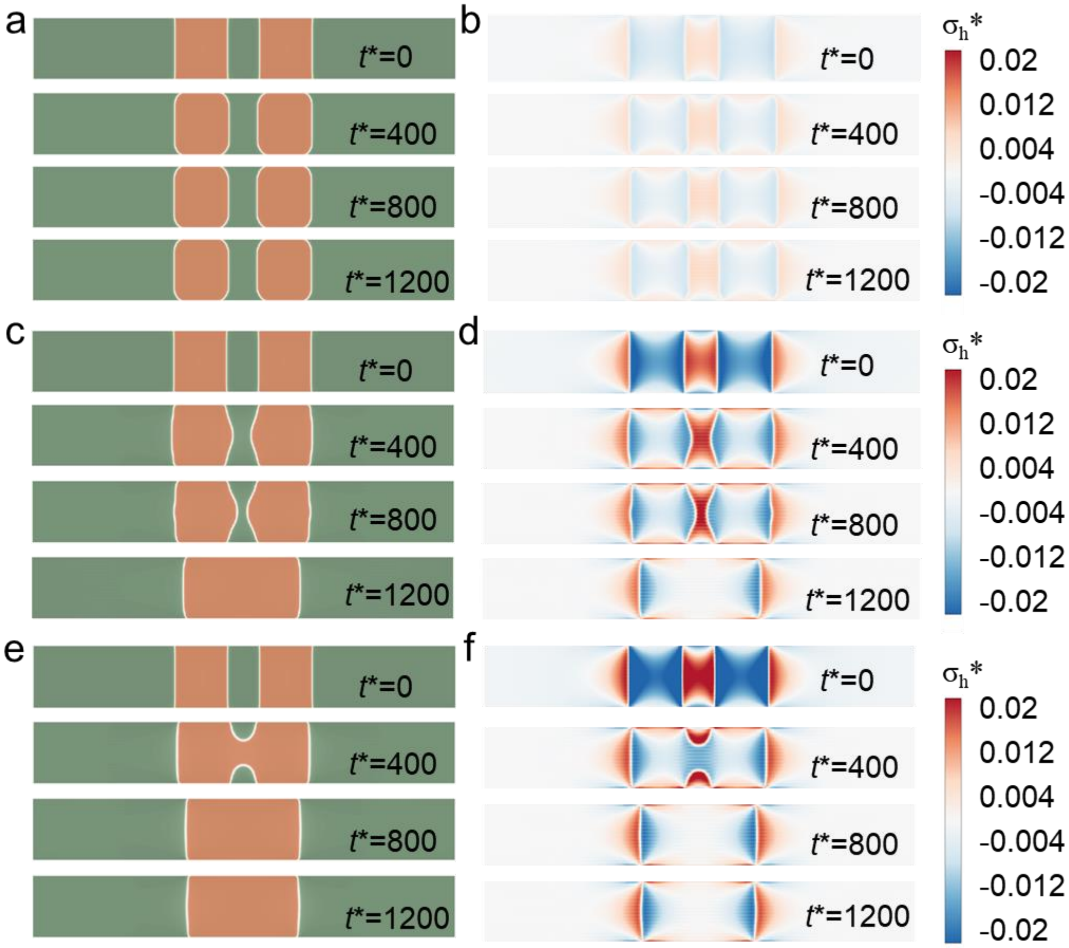

Supplementary Figure 22 | Effect of mismatch strain on the interaction process between two segments at different dimensionless times (t^*=0, 400, 800, 1200). a, b, The morphology of Ag_2Te island and the corresponding distribution of dimensionless hydrostatic stress when the mismatch strain is set as 0.005. **c, d,** The morphology of Ag_2Te island and the corresponding distribution of dimensionless hydrostatic stress when the mismatch strain is set as 0.025. **e, f,** The morphology of Ag_2Te island and the corresponding distribution of dimensionless hydrostatic stress when the mismatch strain is set as 0.045.

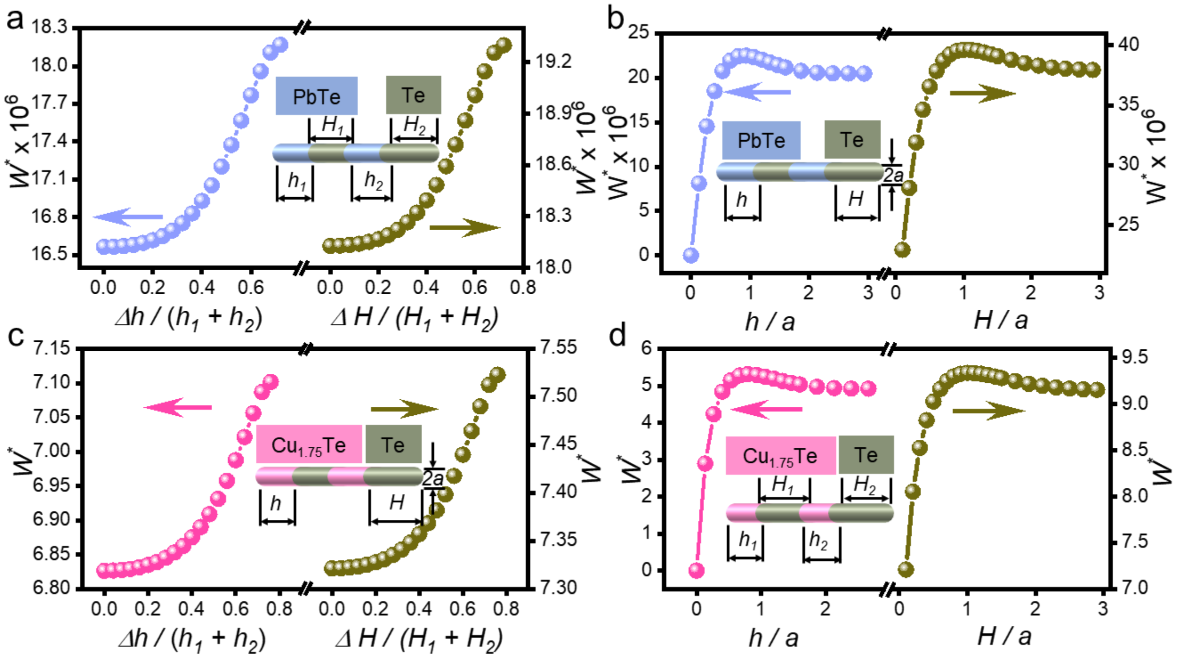

Supplementary Figure 23 | Simulated results of Te/PbTe and Te/$Cu_{1.75}$Te SHs. a, b, The calculated dimensionless elastic energy *versus* the differences in length between adjacent segments in Te/PbTe SHs, segment length and segment separation. **c, d,** The calculated dimensionless elastic energy *versus* the differences in length between adjacent segments in Te/$Cu_{1.75}$Te SHs, segment length and segment separation. Source data are provided as a Source Data file.

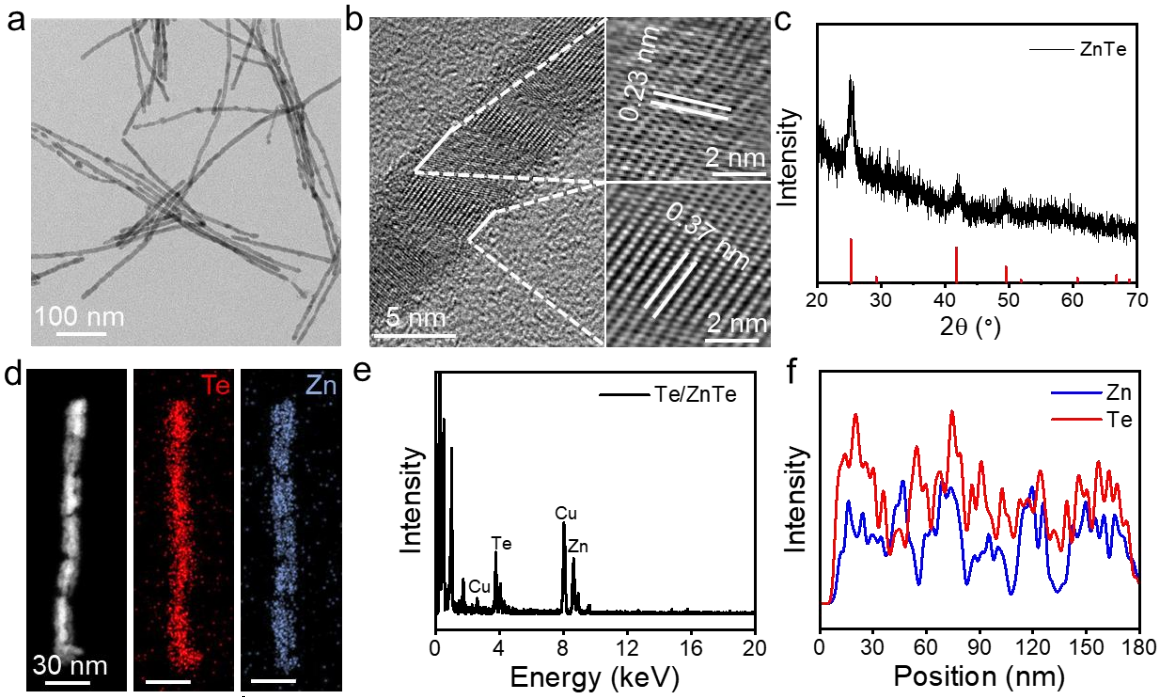

Supplementary Figure 24 | Morphological and structural characterizations of Te/ZnTe SHs. a, b, TEM and HRTEM images, showing the spacings of 0.38 and 0.23 nm corresponding to Te and ZnTe, respectively. **c,** XRD pattern. **d,** HADDF-STEM and elemental mapping characterizations. All scale bar are 30 nm. **e,** EDS. **f,** Line mapping profiles. Source data are provided as a Source Data file.

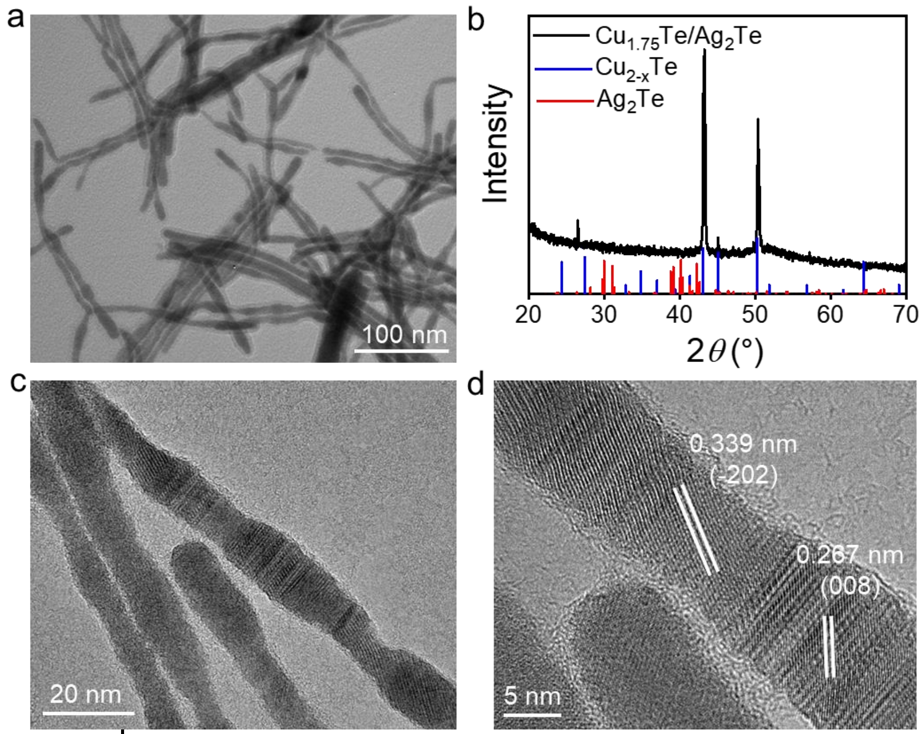

Supplementary Figure 25 │ Morphological and structural characterizations of $Cu_{1.75}Te/Ag_2Te$ SHs. a, TEM image. **b,** XRD pattern. **c, d,** HRTEM images, showing the spacings of 0.339 and 0.267 nm corresponding to Ag_2Te and $Cu_{1.75}Te$, respectively. Source data are provided as a Source Data file.

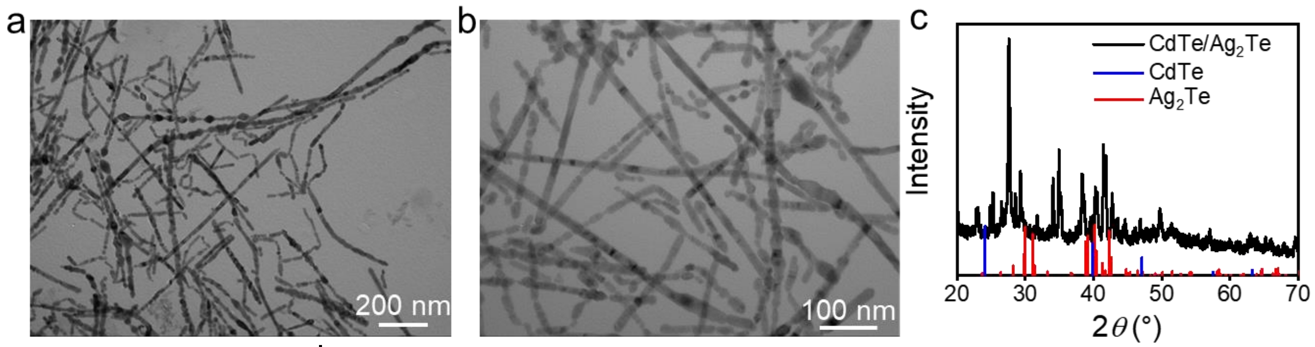

Supplementary Figure 26 │ Morphological and structural characterizations of $CdTe/Ag_2Te$ SHs. a, b, TEM images. **c,** XRD pattern. Source data are provided as a Source Data file.

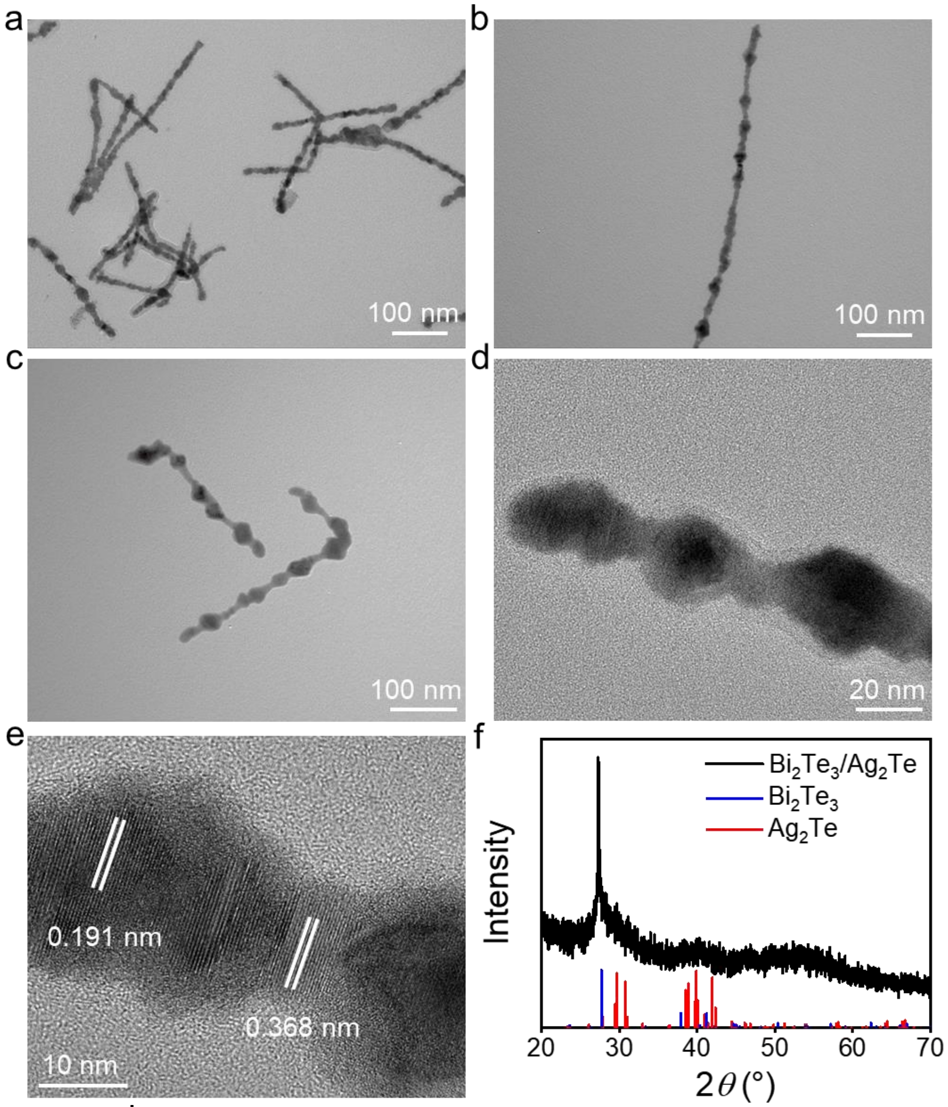

Supplementary Figure 27 │ Morphological and structural characterizations of Bi_2Te_3/Ag_2Te SHs. a, b, c, d, TEM images. **e,** HRTEM image, showing the spacings of 0.191 and 0.368 nm corresponding to Bi_2Te_3 and A_2Te, respectively. **f,** XRD pattern. Source data are provided as a Source Data file.

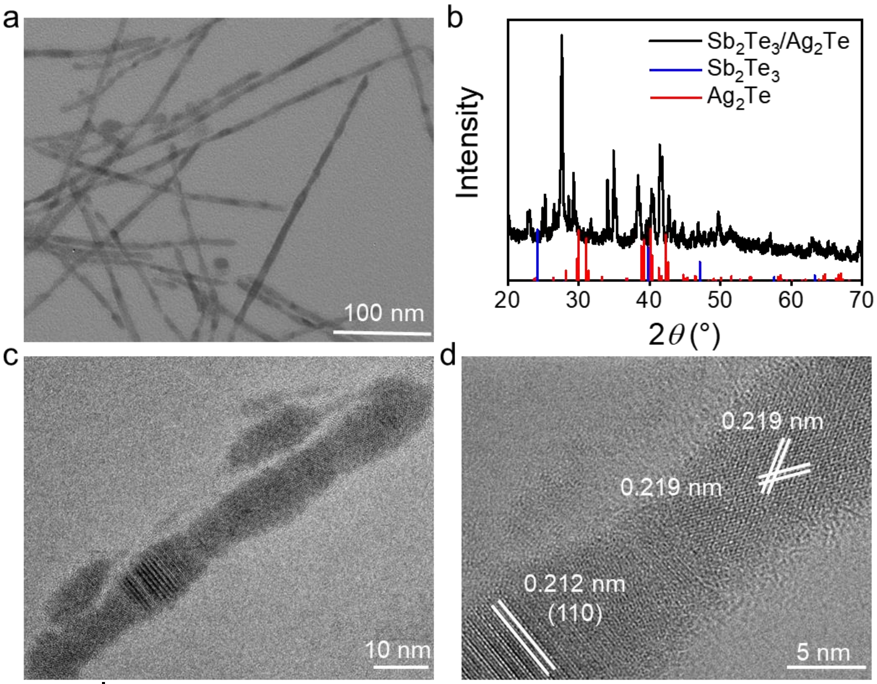

Supplementary Figure 28│Morphological and structural characterizations of Sb_2Te_3/Ag_2Te SHs. a, TEM image. **b,** XRD pattern. **c, d,** HRTEM images, showing the spacings of 0.212 and 0.219 nm corresponding to Sb_2Te_3 and Ag_2Te, respectively. Source data are provided as a Source Data file.

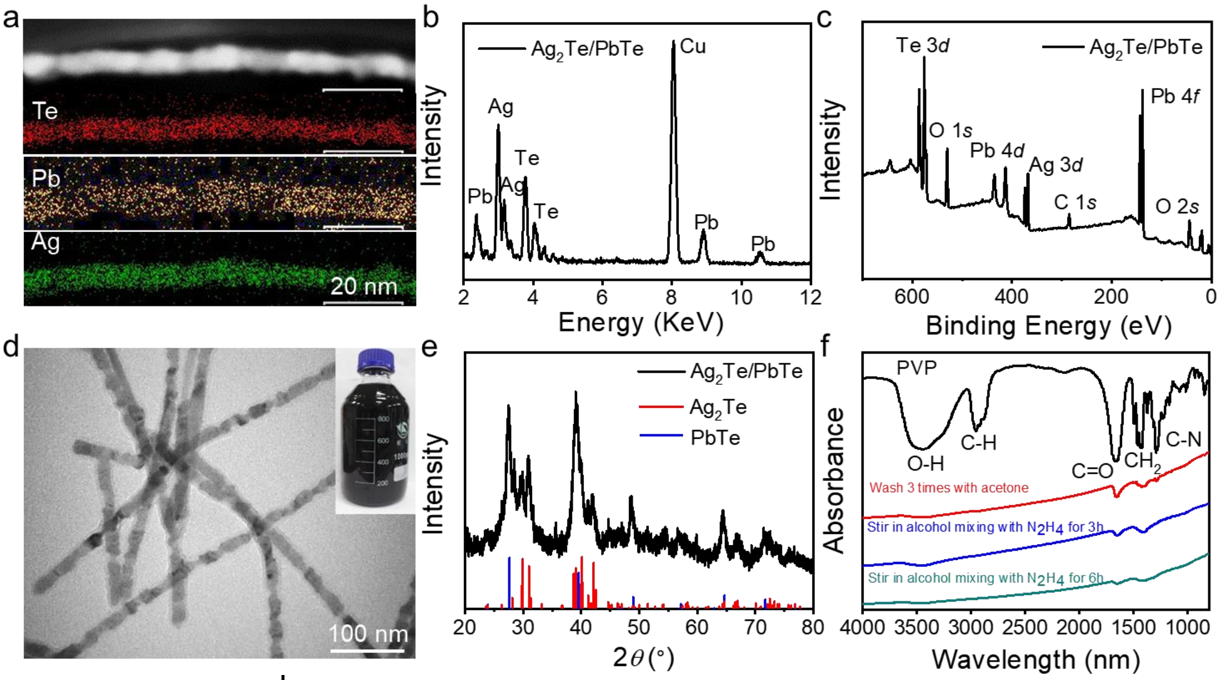

Supplementary Figure 29│Morphological and structural characterizations of $Ag_2Te/PbTe$ SHs. a, HADDF-STEM and elemental mapping characterization. All scale bar are 20 nm. **b,** EDS. **c,** The survey XPS spectra. **d,** TEM image of $Ag_2Te/PbTe$ synthesized at large scale. Inset: One liter of high-quality SH dispersion. **e**, XRD pattern of the SHs synthesized at large scale. **f,** FTIR spectra of the SHs washed with different methods, showing the surfactant PVP can hardly be detected after stirred in alcohol mixing with N_2H_4 for 6h. Source data are provided as a Source Data file.

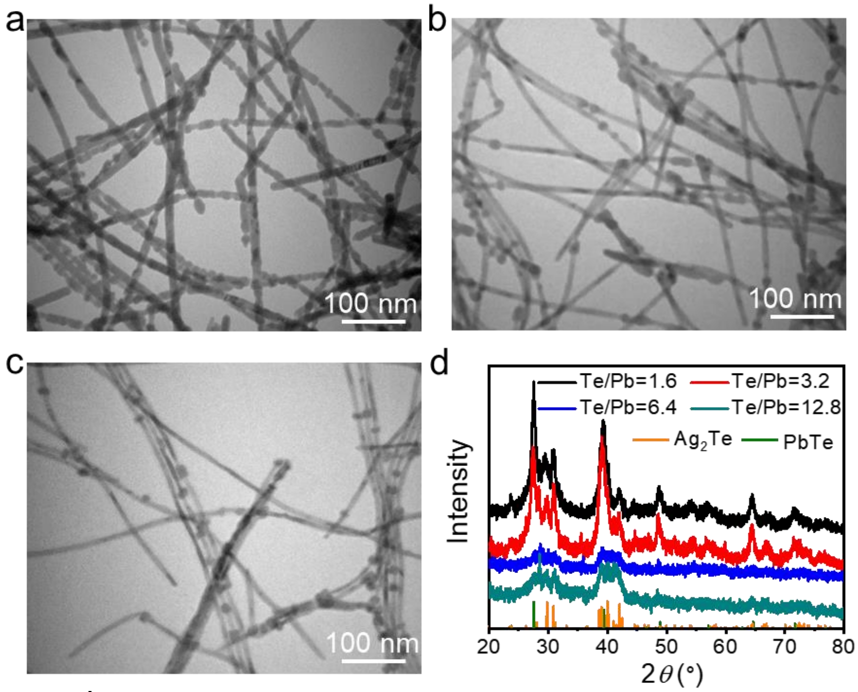

Supplementary Figure 30 │Morphological and structural characterizations of Ag_2Te/PbTe SHs. a, b, c, TEM images with Te/Pb=1.6, 6.4, 12.8. As Pb^{2+} reduces, the proportion of PbTe segments reduces. Correspondingly, Ag_2Te segments transforming from Te increase. **d,** XRD pattern, showing the peak at 27.5° attributed to PbTe weakens accompanied by the peak at 40.1° attributed to Ag_2Te strengthening as the Pb precursor reduces. Source data are provided as a Source Data file.

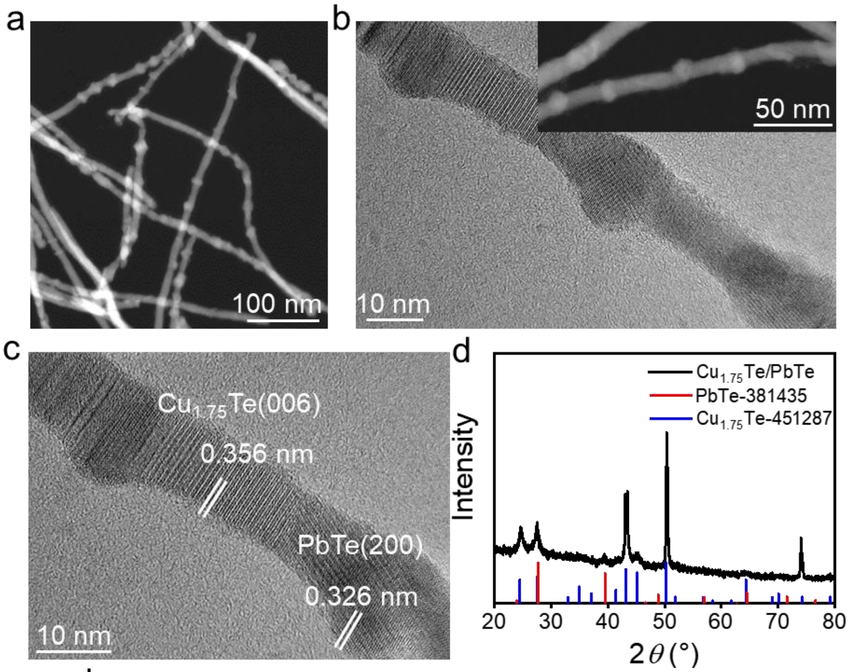

Supplementary Figure 31│Morphological characterizations of $Cu_{1.75}Te$/PbTe SHs. a, HADDF-STEM image. **b,** HRTEM image. Inset: HADDF-STEM image of a single NW. **c,** HRTEM image, showing the spacings of 0.356 and 0.326 nm, corresponding to $Cu_{1.75}Te$ and PbTe, respectively. **d,** XRD pattern, showing the obvious biphases of PbTe and $Cu_{1.75}Te$. Source data are provided as a Source Data file.

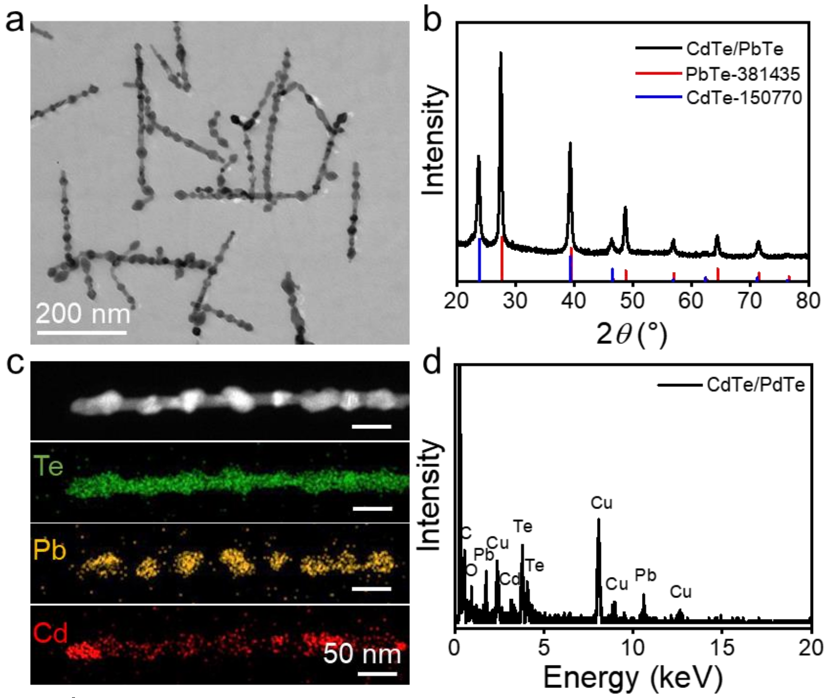

Supplementary Figure 32 │ Morphological characterizations of CdTe/PbTe SHs. a, TEM image. **b,** XRD pattern, showing the coexistence of PbTe and CdTe. **c,** HADDF-STEM and elemental mapping characterization. All scale bars are 50 nm. **d,** EDS. Source data are provided as a Source Data file.

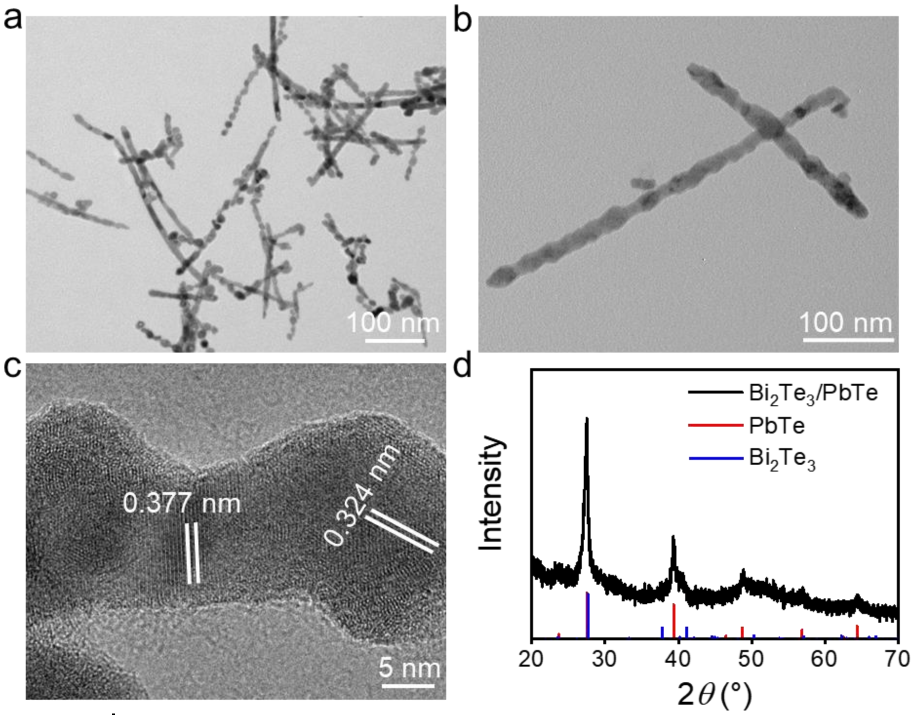

Supplementary Figure 33 │ Morphological characterizations of Bi_2Te_3/PbTe SHs. a, b, TEM images. **c,** HRTEM image, showing the spacings of 0.377 and 0.324 nm, corresponding to Bi_2Te_3 and PbTe, respectively. **d,** XRD pattern, showing the coexistence of PbTe and Bi_2Te_3 phases. Source data are provided as a Source Data file.

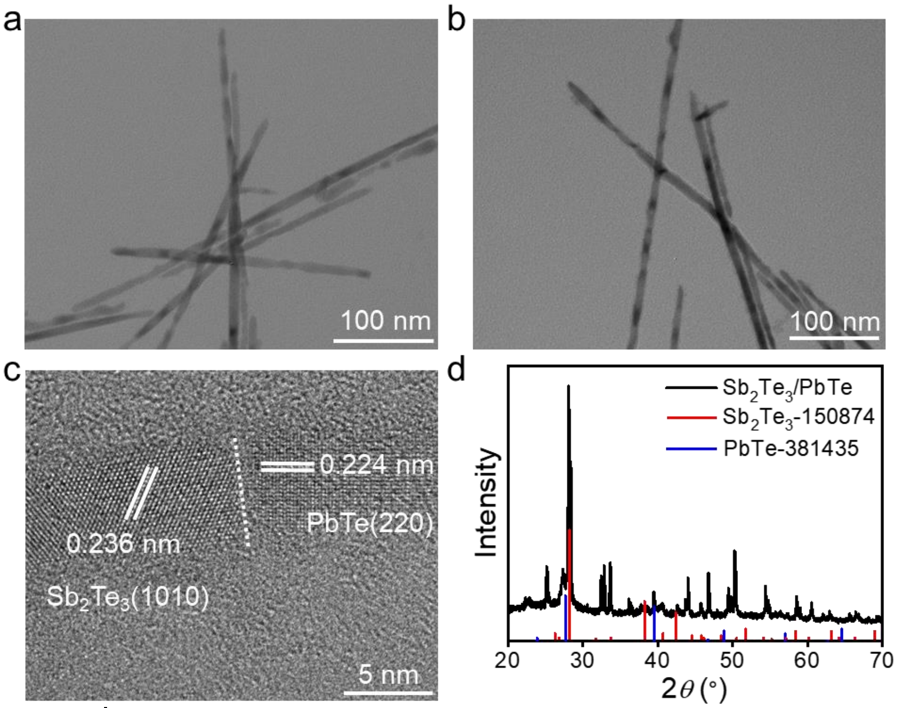

Supplementary Figure 34 │ Morphological characterizations of Sb_2Te_3/PbTe SHs. a, b, TEM images. **c,** HRTEM image, showing the spacings of 0.236 and 0.224 nm, corresponding to Sb_2Te_3 and PbTe, respectively. **d,** XRD pattern, showing the coexistence of PbTe and Sb_2Te_3 phases. Source data are provided as a Source Data file.

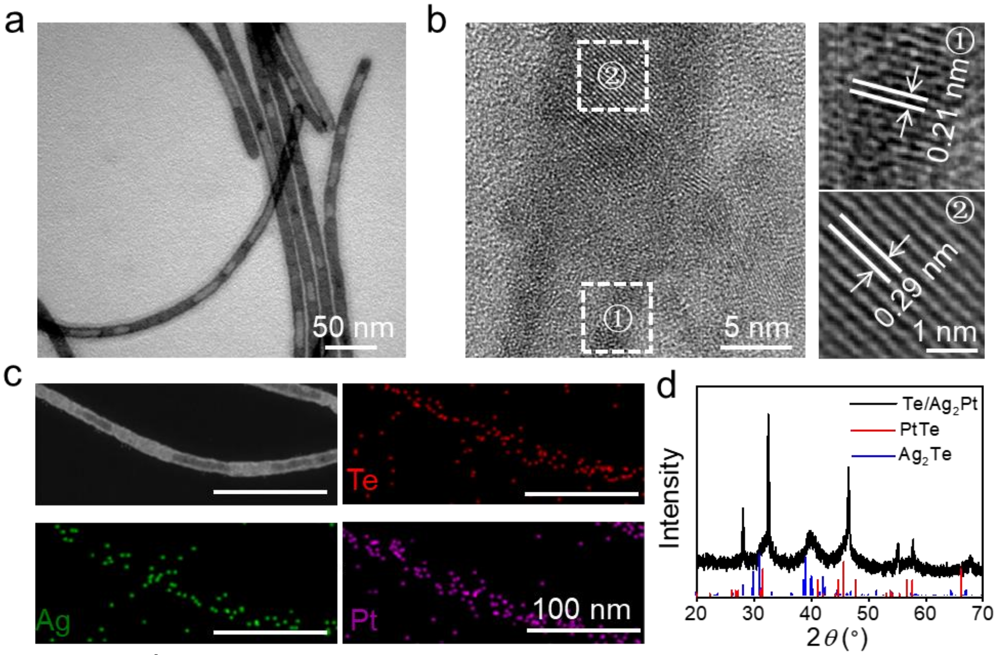

Supplementary Figure 35 │ Morphological and structural characterizations of Pt/Ag_2Te NW-NT SHs. a, TEM image, showing NW and NT distribute alternately. **b,** HRTEM images showing the spacings of 0.21 and 0.29 nm corresponding to PtTe and Ag_2Te, respectively. **c,** HADDF-STEM and elemental mapping characterization. All scale bar are 100 nm. **d,** XRD pattern, showing the coexistence of PtTe and Ag_2Te phases. Source data are provided as a Source Data file.

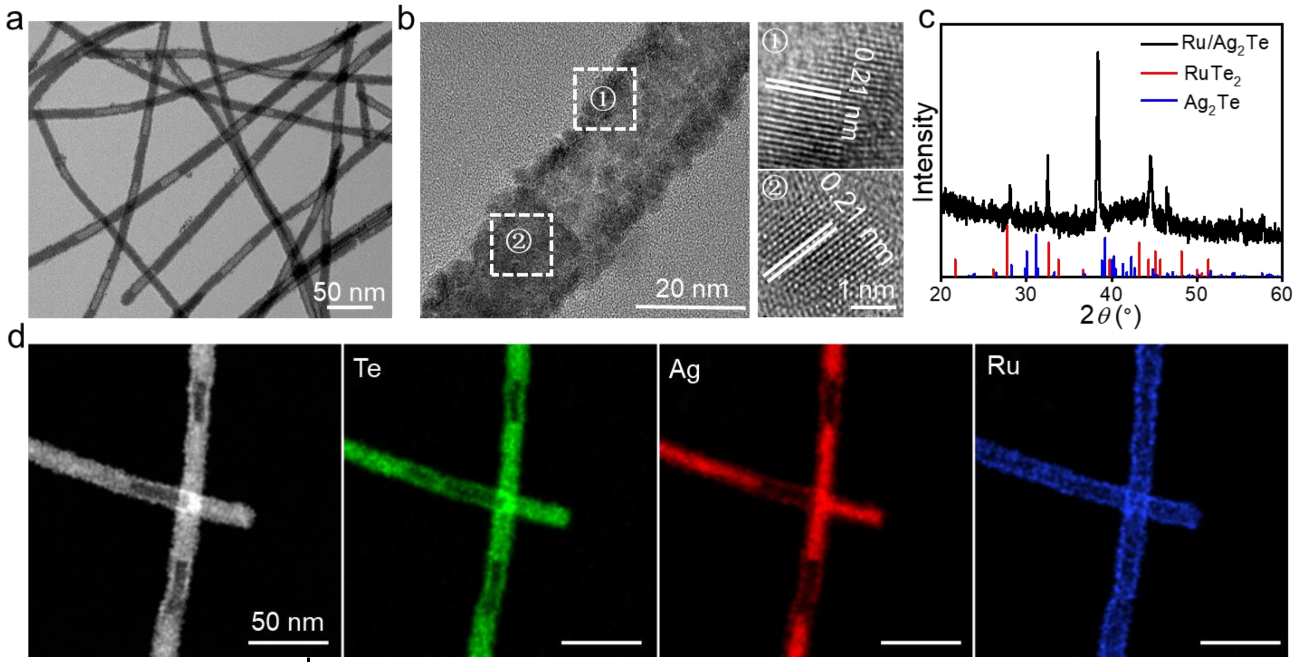

Supplementary Figure 36 | Morphological and structural characterizations of Ru/Ag_2Te NW-NT SHs. **a,** TEM image. **b,** HRTEM images. **c,** XRD pattern, showing the coexistence of $RuTe_2$ and Ag_2Te phases. **d,** HADDF-STEM and elemental mapping characterizations. All scale bar are 50 nm. Source data are provided as a Source Data file.

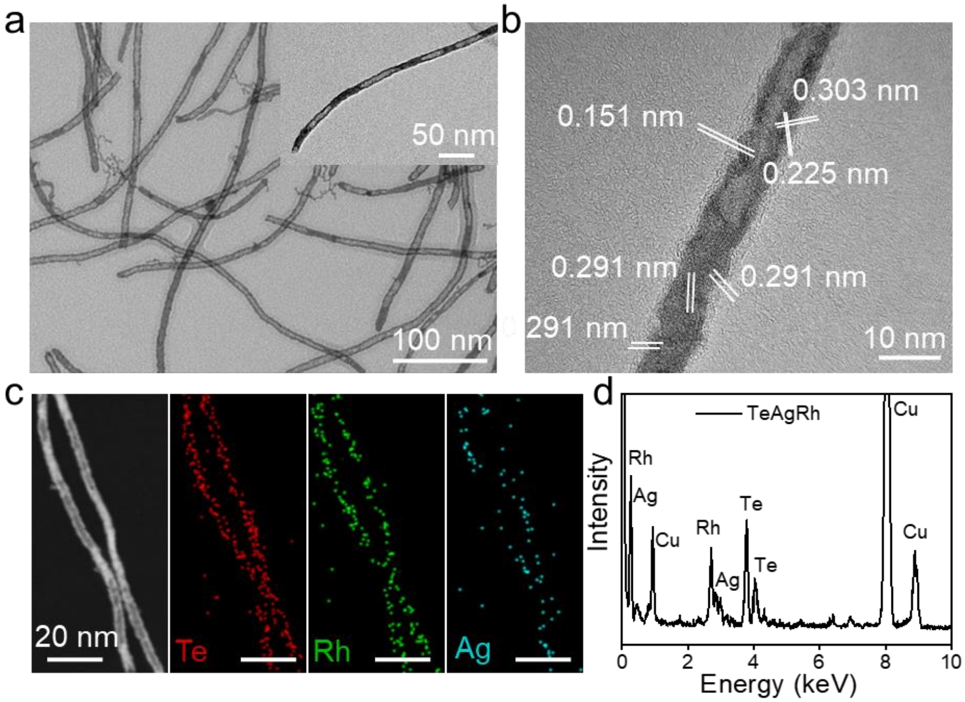

Supplementary Figure 37 | Morphological and structural characterizations of Rh/Ag_2Te NW-NT SHs. **a,** TEM image. **b,** HRTEM image, showing the spacings of 0.225 and 0.291 nm corresponding to RhTe and Ag_2Te, respectively. **c,** HADDF-STEM and elemental mapping characterizations. All scale bars are 20 nm. **d,** EDS. Source data are provided as a Source Data file.

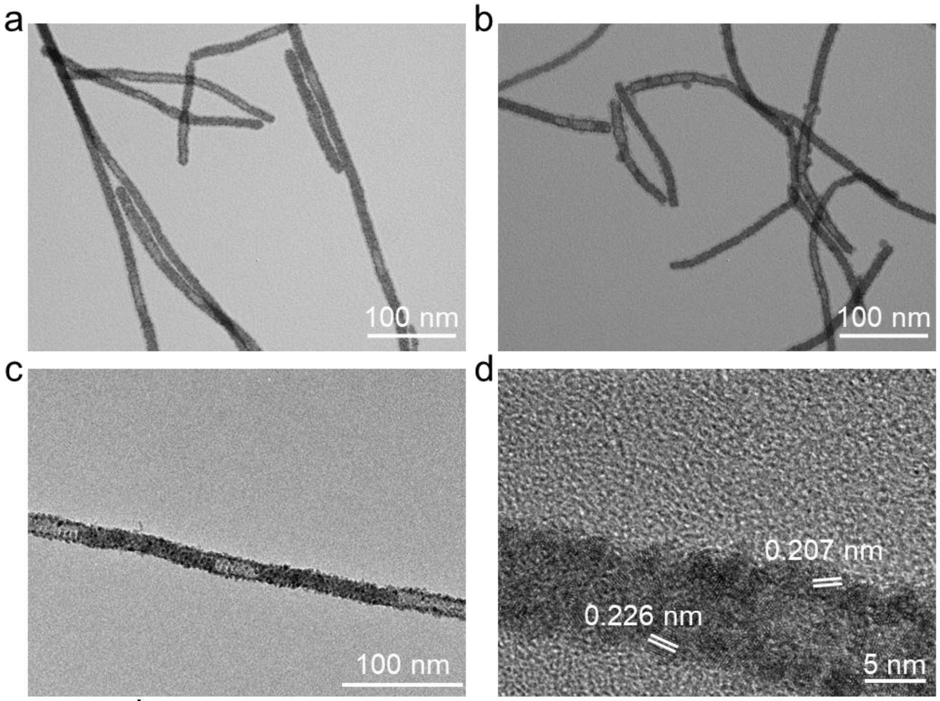

Supplementary Figure 38 | Morphological characterizations of Ir/Ag_2Te NW-NT SHs. a, b, c, TEM images. **d,** HRTEM image.

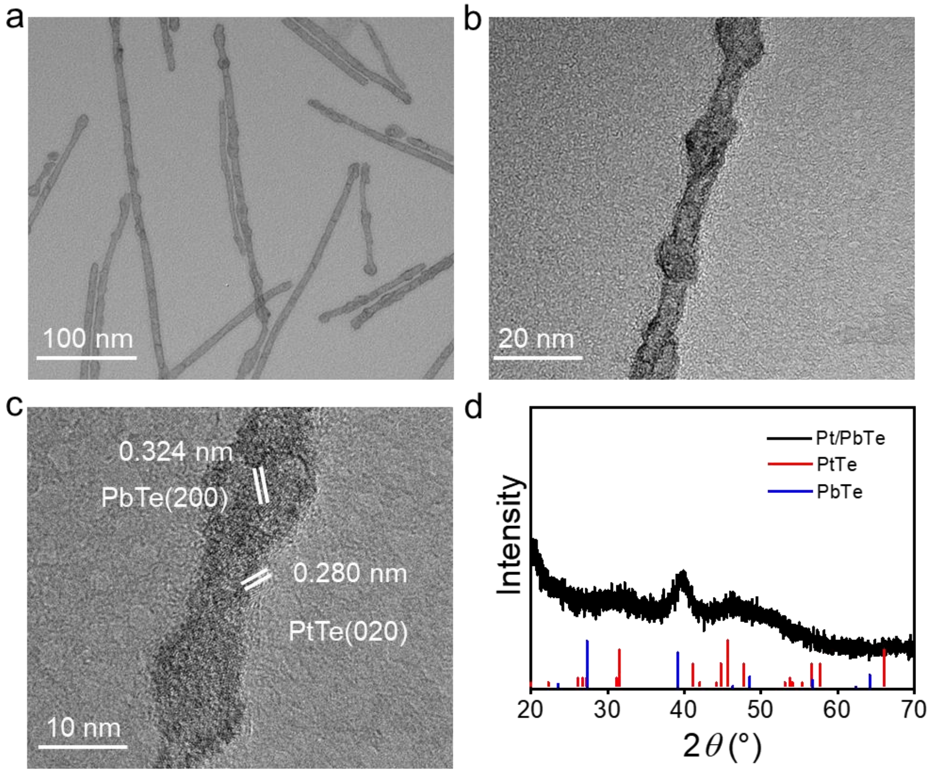

Supplementary Figure 39 | Morphological and structural characterizations of Pt/PbTe NW-NT SHs. a, b, TEM images. **c,** HRTEM image, showing the spacings of 0.280 and 0.324 nm, corresponding to PtTe at the NT and PbTe at the node, respectively. **d,** XRD pattern, showing the coexistence of PbTe and PtTe. Source data are provided as a Source Data file.

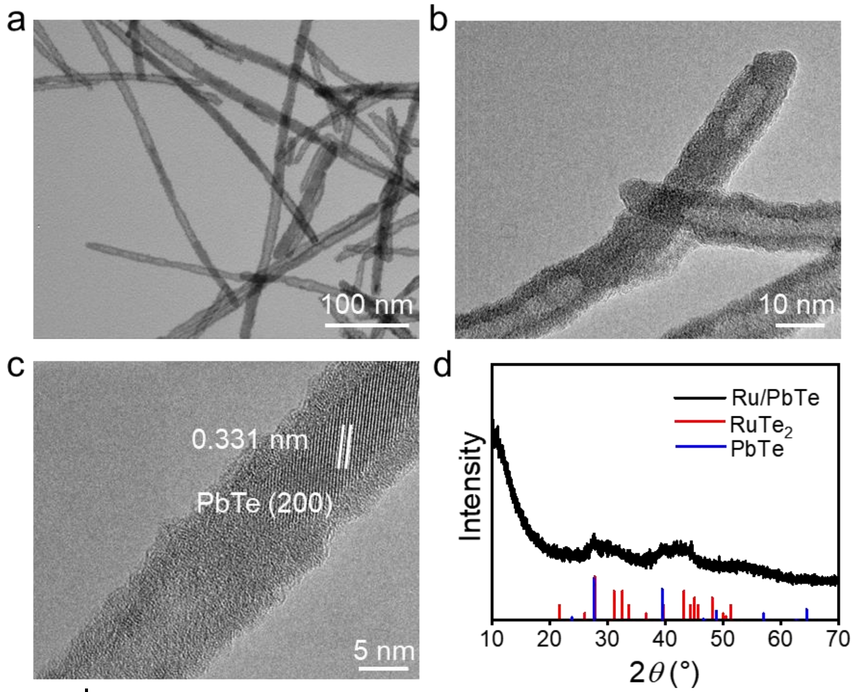

Supplementary Figure 40 | Morphological and structural characterizations of Ru/PbTe NW-NT SHs. a, TEM image. **b, c,** HRTEM images, showing the spacings of 0.331 and 0.324 nm, corresponding to PbTe and $RuTe_2$, respectively. **d,** XRD pattern, showing the coexistence of PbTe and $RuTe_2$. Source data are provided as a Source Data file.

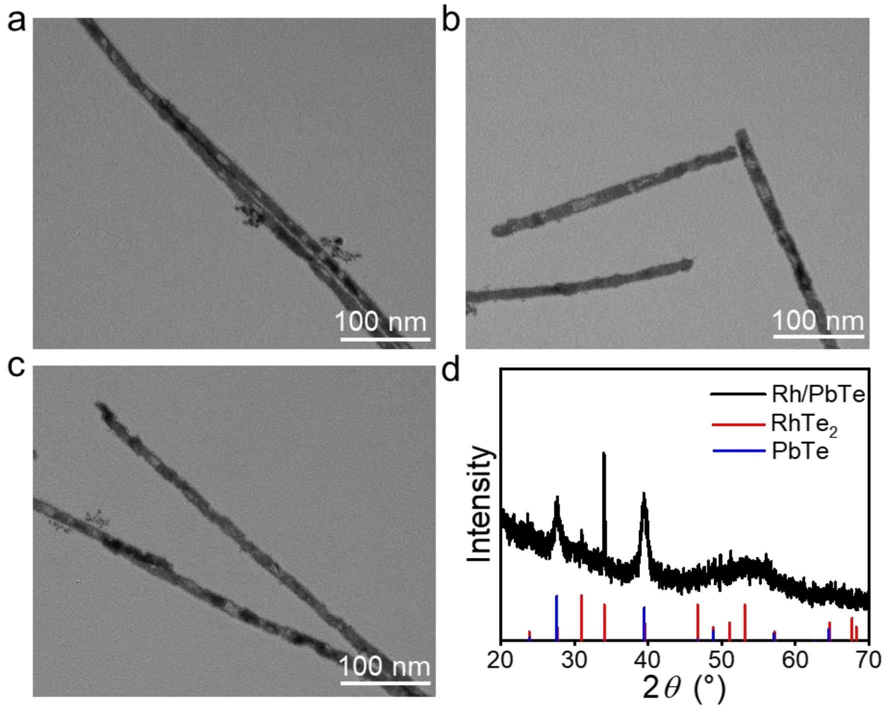

Supplementary Figure 41 | Morphological and structural characterizations of Rh/PbTe NW-NT SHs. a, b, c, TEM images. **d,** XRD pattern, showing the coexistence of PbTe and $RhTe_2$. Source data are provided as a Source Data file.

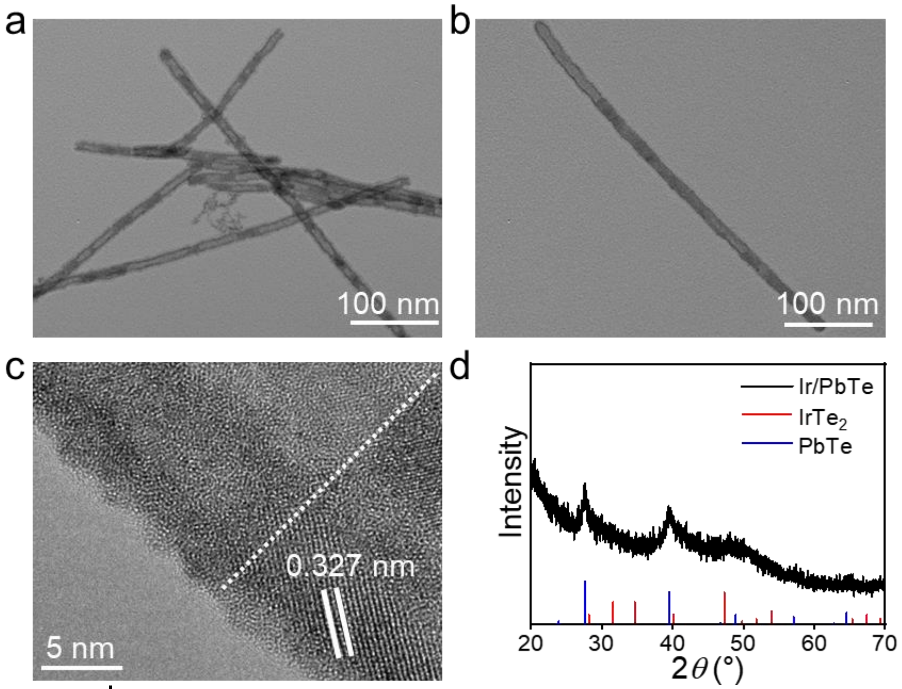

Supplementary Figure 42 │ Morphological and structural characterizations of Ir/PbTe NW-NT SHs. a, b, TEM images. **c,** HRTEM image. **d,** XRD pattern, showing the coexistence of PbTe and $IrTe_2$. Source data are provided as a Source Data file.

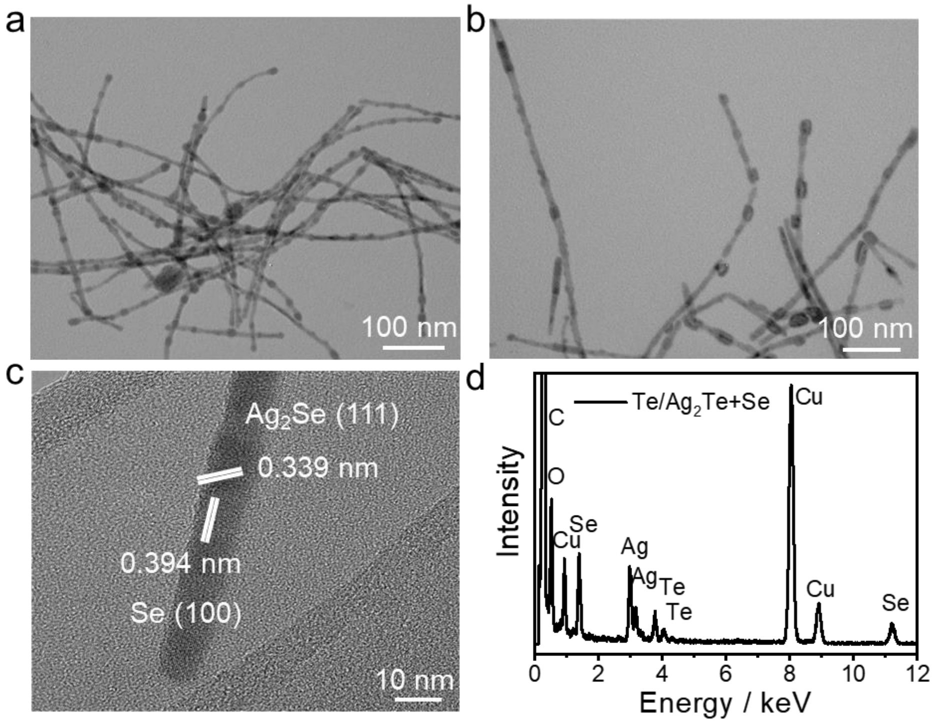

Supplementary Figure 43 │ Morphological and structural characterizations of TeSe/AgTeSe SHs. a, b, TEM images. **c,** HRTEM image, showing the alternative spacings of 0.394 and 0.339 nm, corresponding to Se and Ag_2Se, respectively. **d,** EDS. Source data are provided as a Source Data file.

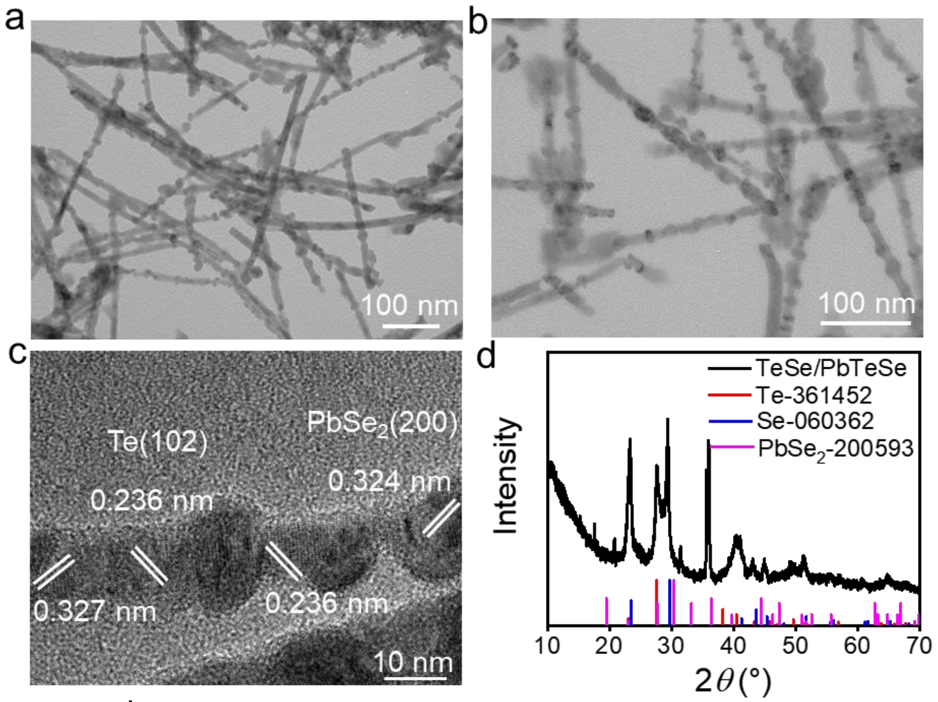

Supplementary Figure 44 | Morphological and structural characterizations of TeSe/PbTeSe SHs. a, b, TEM images. **c,** HRTEM image, showing the alternative spacings of 0.236 and 0.324 nm, corresponding to Te and PbSe, respectively. **d,** XRD pattern. Source data are provided as a Source Data file.

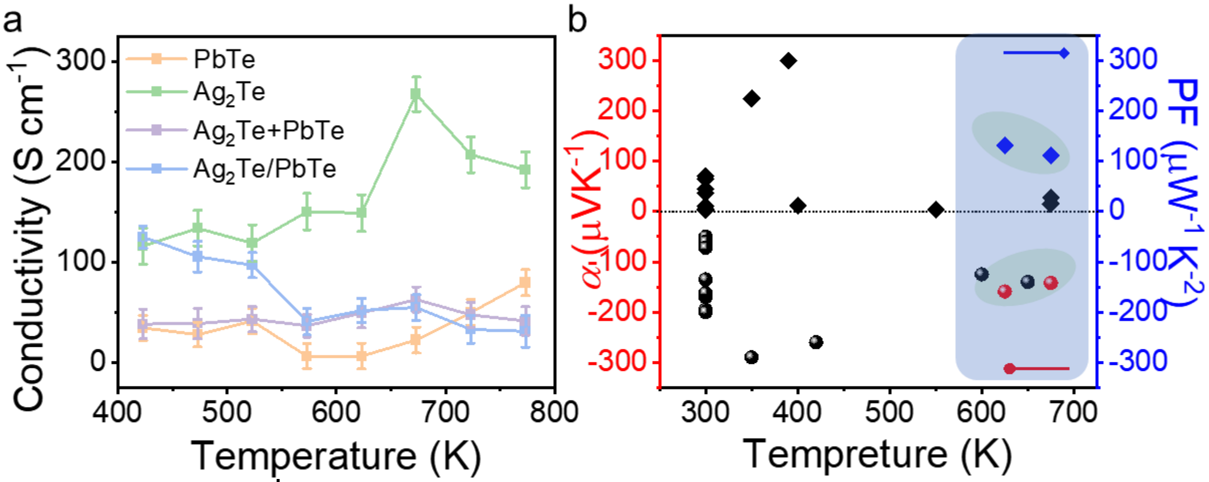

Supplementary Figure 45 | The thermoelectric performances of Ag_2Te/PbTe 1D SHs. a, Electrical conductivity. Error bars correspond to the standard deviations taken over three measurements. **b,** Seebeck coefficient and power factor comparison among different reported PbTe-based materials. Source data are provided as a Source Data file.

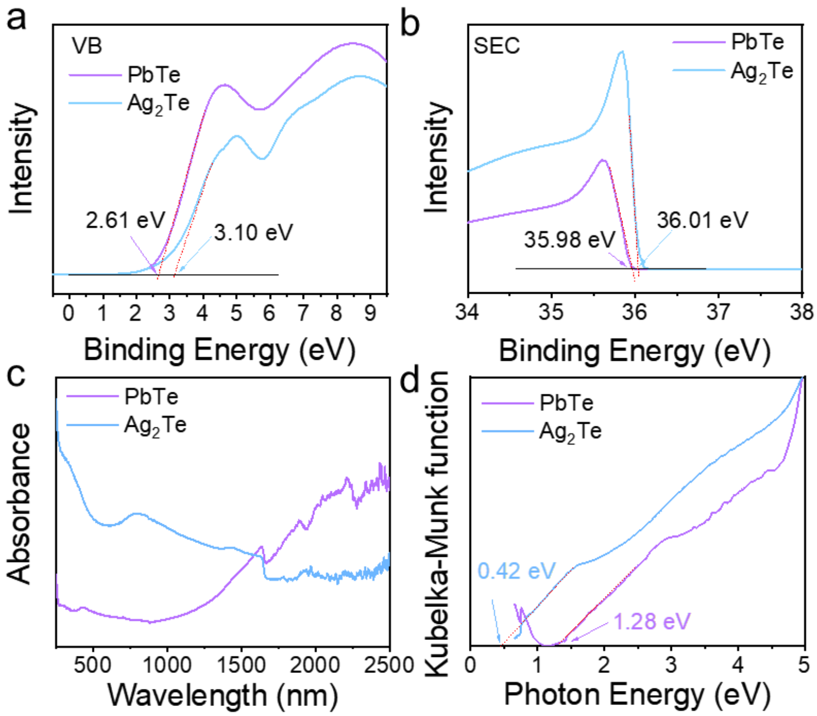

Supplementary Figure 46│Electronic and spectral structures of PbTe and Ag_2Te NWs measured by Synchrotron radiation photoemission spectroscopy (SRPES). a, Valence-band spectra. **b,** Secondary electron cutoff. hν=40.15 eV. **c,** UV−vis−IR diffuse reflectance spectra. **d,** The converted Kubelka-Munk function. Source data are provided as a Source Data file.

Supplementary Table 1 | Compositions of Te/Ag_2Te SHs determined by ICP.

**Sample**	**1**	**2**	**3**	**4**	**5**
Feed ratios (Te/Ag)	1:0	40:1	10:1	4:1	0.5:1
ICP results (atomic ratios)	Ag_0Te_1	$Ag_{0.026}Te_{0.974}$	$Ag_{0.123}Te_{0.877}$	$Ag_{0.195}Te_{0.805}$	$Ag_{0.659}Te_{0.341}$

Supplementary Table 2 | Values of material parameters.

Parameters	Symbols	Values	References
Young's modulus of Te	E_{Te}	22 GPa	1, 2
Poisson's ratio of Te	V_{Te}	0.22	1, 2
Young's modulus of Ag_2Te	$E_{\mathrm{Ag_2Te}}$	54 GPa	1, 2
Poisson's ratio of Ag_2Te	$V_{\mathrm{Ag_2Te}}$	0.37	1, 2
Young's modulus of $Cu_{1.75}Te$	$E_{\mathrm{Cu_{1.75}Te}}$	82 GPa	1, 2
Poisson's ratio of $Cu_{1.75}Te$	$V_{\mathrm{Cu_{1.75}Te}}$	0.3	1, 2
Young's modulus of PbTe	E_{PbTe}	40 GPa	1, 2
Poisson's ratio of PbTe	V_{PbTe}	0.25	1, 2
Temperature	T	300 K	-
Enthalpy of mixing	ω	0.0259 eV	-
Boltzmann constant	$\mathrm{k_B}$	1.38×10^{-23} J K^{-1}	-
Number of Ag^+ per unit volume	ρ	1.81×10^{24} m^{-3}	-
Number of Cu^{2+} per unit volume		4.03×10^{24} m^{-3}	-
Number of Pb^{2+} per unit volume		6.26×10^{24} m^{-3}	-
Expansion coefficient of Te-Ag_2Te	β	0.035	-
Expansion coefficient of Te-$Cu_{1.75}Te$		0.009	-
Expansion coefficient of Te-PbTe		0.027	-
Diffusivity of Te-Ag_2Te	D	5.0×10^{-10} cm^2 s^{-1}	3
Diffusivity of Te-$Cu_{1.75}Te$		1.1×10^{-10} cm^2 s^{-1}	4
Diffusivity of Te-PbTe		5.6×10^{-10} cm^2 s^{-1}	5
Gradient coefficient	κ	2×10^{-9} J m^{-1}	6

Supplementary Movie 1 | In-situ TEM observation of the island formation.

Supplementary Movie 2 | Three-stage evolution process of 1D segmented heterogeneous nanostructures.

Supplementary Movie 3 | Ordering enabled by the stress induced ordering mechanism.

Supplementary References

1 Maarten, D. J., *et al.*, Charting the complete elastic properties of inorganic crystalline compounds. *Sci. Data* **2**, 150009 (2015).

2 J. A *et al.*, The Materials Project: A materials genome approach to accelerating materials innovation. *APL Materials* **1**, 011002 (2013).

3 Takahash, T., Solid ionics-solid electrolyte cells. *J. Electrochem. Soc.* **117**, 1 (1970).

4 Bucur, R. V., R. Berger, Electrochemical potentiometric determination of the diffusion coefficient of copper in low digenite, a copper sulfide. *Solid State Ionics* **76**, 291 (1995).

5. Brady, E. L., Preparation and properties of lead telluride. *J. Electrochem. Soc.* **101**, 466 (1954).

6. Chen, L., *et al.*, A Phase-field model coupled with large elasto-plastic deformation: application to lithiated silicon electrodes. *J. Electrochem. Soc.* **161**, 3164 (2014).
