# Supplementary material for: Stress-induced ordering evolution of 1D segmented heteronanostructures and their chemical post-transformations
Source: Nat Commun. 2024 Apr 13;15:3208. doi: 10.1038/s41467-024-47446-7 (PMC11271508; doi:10.1038/s41467-024-47446-7)
Supplement: Supplementary file 3 — Description of Additional Supplementary Files [file 41467_2024_47446_MOESM3_ESM.pdf]

Description of Additional Supplementary Files

File Name: Supplementary Data 1
Description: Detailed reaction conditions for the synthesis of SHs.

File Name: Supplementary Movie 1
Description: In-situ TEM observation of the island formation.

File Name: Supplementary Movie 2
Description: Threestage evolution process of 1D segmented heterogeneous nanostructures.

File Name: Supplementary Movie 3
Description: Ordering enabled by the stress induced ordering mechanism.
