# Supplementary material for: Stress-induced ordering evolution of 1D segmented heteronanostructures and their chemical post-transformations
Source: Nat Commun. 2024 Apr 13;15:3208. doi: 10.1038/s41467-024-47446-7 (PMC11271508; doi:10.1038/s41467-024-47446-7)
Supplement: Supplementary file 4 — Supplementary Data 1 [file 41467_2024_47446_MOESM4_ESM.pdf]

Supplementary Data 1 | Detailed reaction conditions for the synthesis of SHs.

Segmented structures	Template	Synthetic method	Solvent	Temperature (ºC)	Comments
**Te/Ag_2Te**	Te	Magnetic stirring	EG	R.T.	Adding NH_4SCN to derate; Ag source: $AgNO_3$
**Te/PbTe**	Te	Hydrothermal synthesis	H_2O	100	Pb source: $PbNO_3$
**Te/$Cu_{1.75}$Te**	Te	Magnetic stirring	EG	R.T.	Reductant: Vc; Cu source: $Cu(NO_3)_2{\cdot}3H_2O$
**Te/Bi_2Te_3**	Te	Temperature programming	TEG	200	10 ºC min^{-1}, for 20 min; pH=10; Bi source: $Bi(NO_3)_2{\cdot}5H_2O$
**Te/CdTe**	Te	Hydrothermal synthesis	H_2O	140	Cd source: $CdCl_2{\cdot}2.5H_2O$
**$Cu_{1.75}$Te/Ag_2Te**	Te/Ag_2Te	Magnetic stirring	H_2O	R.T.	Reductant: Vc; Cu source: $Cu(NO_3)_2{\cdot}3H_2O$
**CdTe/Ag_2Te**	Te/Ag_2Te	Hydrothermal synthesis	H_2O	140	Cd source: $CdCl_2{\cdot}2.5H_2O$
**Bi_2Te_3/Ag_2Te**	Te/Ag_2Te	Temperature programming	TEG	200	10 ºC min^{-1}, for 20 min; pH=10; Bi source: $Bi(NO_3)_2{\cdot}5H_2O$
**ZnTe/Ag_2Te**	Te/Ag_2Te	Water bath	CH_3OH	50	Reductant: TBP; Zn source: $Zn(NO_3)_2$
**Sb_2Te_3/Ag_2Te**	Te/Ag_2Te	Magnetic stirring	EG	R.T.	Reductant: Vc; Sb source: $Sb(CH_3COO)_3$
**TeSe/AgTeSe**	Te/Ag_2Te	Water bath	H_2O	80	Reductant: $N_2H_4{\cdot}H_2O$; Se source: Se powder
**Pt/Ag_2Te**	Te/Ag_2Te	Shaking table	EG	160	Pt source: H_2PtCl_6
**Ru/Ag_2Te**	Te/Ag_2Te	Solvothermal synthesis	EG	160	Ru source: $RuCl_3{\cdot}H_2O$
**Rh/Ag_2Te**	Te/Ag_2Te	Solvothermal synthesis	EG	160	Rh source: $RhCl_3{\cdot}H_2O$
**Ir/Ag_2Te**	Te/Ag_2Te	Solvothermal synthesis	EG	160	Ir source: $IrCl_3{\cdot}H_2O$
**Ag_2Te/PbTe**	Te/PbTe	Magnetic stirring	EG	R.T.	Ag source: $AgNO_3$
**Bi_2Te_3/PbTe**	Te/PbTe	Temperature programming	TEG	200	10ºC min^{-1}, for 20min; pH=10; Bi source: $Bi(NO_3)_2{\cdot}5H_2O$

**$Cu_{1.75}Te$/PbTe**	Te/PbTe	Magnetic stirring	EG	R.T.	Reductant: Vc;<br>Cu source: $Cu(NO_3)_2 \cdot 3H_2O$
**CdTe/PbTe**	Te/PbTe	Hydrothermal synthesis	H_2O	140	Cd source: $CdCl_2 \cdot 2.5H_2O$
**Sb_2Te_3/PbTe**	Te/PbTe	Magnetic stirring	EG	R.T.	Reductant: Vc;<br>Sb source: $Sb(CH_3COO)_3$
**TeSe/PbTeSe**	Te/PbTe	Water bath	H_2O	80	Reductant: $N_2H_4 \cdot H_2O$;<br>Se source: Se powder
**Pt/PbTe**	Te/PbTe	Shaking table	EG	160	Pt source: H_2PtCl_6
**Ru/PbTe**	Te/PbTe	Solvothermal synthesis	EG	160	Ru source: $RuCl_3 \cdot H_2O$
**Rh/PbTe**	Te/PbTe	Solvothermal synthesis	EG	160	Rh source: $RhCl_3 \cdot H_2O$
**Ir/PbTe**	Te/PbTe	Solvothermal synthesis	EG	160	Ir source: $IrCl_3 \cdot H_2O$
